# Walking pathways with positive feedback loops reveal DNA methylation biomarkers of colorectal cancer

**DOI:** 10.1186/s12859-019-2687-7

**Published:** 2019-04-18

**Authors:** Alexander Kel, Ulyana Boyarskikh, Philip Stegmaier, Leonid S. Leskov, Andrey V. Sokolov, Ivan Yevshin, Nikita Mandrik, Daria Stelmashenko, Jeannette Koschmann, Olga Kel-Margoulis, Mathias Krull, Anna Martínez-Cardús, Sebastian Moran, Manel Esteller, Fedor Kolpakov, Maxim Filipenko, Edgar Wingender

**Affiliations:** 1 Institute of Chemical Biology and Fundamental Medicine, SBRAN, Novosibirsk, 630090 Russia; 2 Biosoft.ru, Ltd, Novosibirsk, 630090 Russia; 3 geneXplain GmbH, 38302 Wolfenbüttel, Germany; 4 City Clinical Hospital №1, Novosibirsk, 630090 Russia; 5 Cancer Epigenetics and Biology Program (PEBC), Bellvitge Biomedical Research Institute (IDIBELL), 08908 Barcelona, Spain; 6 Centro de Investigacion Biomedica en Red Cancer (CIBERONC), 28029 Madrid, Spain; 7 Physiological Sciences Department, School of Medicine and Health Sciences, University of Barcelona (UB), 08010 Barcelona, Spain; 8 Institucio Catalana de Recerca i Estudis Avançats (ICREA), 08010 Barcelona, Spain; 9 Institute of Computational Technologies SB RAS, Novosibirsk, 630090 Russia; 10 Institute of Bioinformatics, University Medical Center Göttingen (UMG), Göttingen, 37077 Germany

**Keywords:** Prognostic biomarkers, Colorectal cancer, Multi-omics analysis, DNA methylation, Circulating DNA, Transcription factor binding sites, Signal transduction, Genetic algorithm

## Abstract

**Background:**

The search for molecular biomarkers of early-onset colorectal cancer (CRC) is an important but still quite challenging and unsolved task. Detection of CpG methylation in human DNA obtained from blood or stool has been proposed as a promising approach to a noninvasive early diagnosis of CRC. Thousands of abnormally methylated CpG positions in CRC genomes are often located in non-coding parts of genes. Novel bioinformatic methods are thus urgently needed for multi-omics data analysis to reveal causative biomarkers with a potential driver role in early stages of cancer.

**Methods:**

We have developed a method for finding potential causal relationships between epigenetic changes (DNA methylations) in gene regulatory regions that affect transcription factor binding sites (TFBS) and gene expression changes. This method also considers the topology of the involved signal transduction pathways and searches for positive feedback loops that may cause the carcinogenic aberrations in gene expression. We call this method “Walking pathways”, since it searches for potential rewiring mechanisms in cancer pathways due to dynamic changes in the DNA methylation status of important gene regulatory regions (“epigenomic walking”).

**Results:**

In this paper, we analysed an extensive collection of full genome gene-expression data (RNA-seq) and DNA methylation data of genomic CpG islands (using Illumina methylation arrays) generated from a sample of tumor and normal gut epithelial tissues of 300 patients with colorectal cancer (at different stages of the disease) (data generated in the EU-supported SysCol project). Identification of potential epigenetic biomarkers of DNA methylation was performed using the fully automatic multi-omics analysis web service “My Genome Enhancer” (MGE) (my-genome-enhancer.com). MGE uses the database on gene regulation TRANSFAC®, the signal transduction pathways database TRANSPATH®, and software that employs AI (artificial intelligence) methods for the analysis of cancer-specific enhancers.

**Conclusions:**

The identified biomarkers underwent experimental testing on an independent set of blood samples from patients with colorectal cancer. As a result, using advanced methods of statistics and machine learning, a minimum set of 6 biomarkers was selected, which together achieve the best cancer detection potential. The markers include hypermethylated positions in regulatory regions of the following genes: CALCA, ENO1, MYC, PDX1, TCF7, ZNF43.

**Electronic supplementary material:**

The online version of this article (10.1186/s12859-019-2687-7) contains supplementary material, which is available to authorized users.

## Background

Search for molecular biomarkers of colorectal cancer (CRC) is an important and still quite challenging and yet unsolved task, despite extensive studies of many research groups in the world. Especially important are early biomarkers that might recognize either the predisposition or early stage of the disease. Cancer-specific regulation of gene expression by aberrant DNA methylation has been extensively described for CRC. Also, methylated DNA fragments are well represented in the circulating DNA. Therefore, detection of CpG island methylation in human DNA obtained from blood or stool has been proposed as a promising approach for noninvasive screening and early diagnosis of colorectal neoplasms [1, 2]. Aberrantly methylated genomic DNA fragments are considered as attractive biomarkers for cancer detection and diagnosis because of their presence as part of cell-free circulating tumor DNA (ctDNA) in body fluids (liquid biopsies) such as stool and blood. Usually, CRC methylome is characterized by thousands of abnormally methylated CpG positions in genome, often located in non-coding parts of genes [3]. Only few of them actually have a cancer-driving role and their methylation level correlates with cancer-specific changes of expression of the respective genes. With huge amount of “omics” data generated today novel bioinformatics methods are urgently needed that would be able to reveal causative biomarkers with potential driver role on early stage of cancer from a multi-omics data analysis.

Colorectal cancer is one of the best-studied types of cancer, at least in terms of its molecular etiology in comparison with all other types of cancers that originate from epithelial cells. Recently, a great number of studies have been carried out worldwide to decipher the molecular mechanisms of development of this type of cancer. Large international consortia, such as ICGC [4] and SysCol [5] dedicated their work to this goal. These consortia have generated a massive amount of genomic, transcriptomic and epigenomic cancer data. These extremely valuable sources of information have to be mined now to identify molecular markers that can be used for early diagnosis of CRC. Thus, the present work was dedicated to the detailed analysis of large volumes of RNA-seq and DNA methylation data, primarily generated by the SysCol project and published in previous publication [6].

In our study we focused our attention on the identification of DNA methylation events as potential biomarkers of early carcinogenic processes. The successful application of one of the first DNA methylation biomarkers, SEPT9, to detect colorectal cancer is one of the hallmarks in this direction [7]. It became evident that the analysis of DNA methylation events in early stages of cancer and understanding the molecular mechanisms of their influence on the drastic changes of gene expression in cancer will give us a key to identify most promising and robust biomarkers. Analyzing the influence of DNA methylation on the binding of transcription factors (TFs) to the genome is very important to understand the role of DNA methylation in the regulation of gene expression. In a recent publication, Yin et al. have systematically analysed the effect of CpG methylation on the binding of 542 human transcription factors [8]. They found that CpG methylation can significantly inhibit binding of many TFs of such classes as bHLH, bZIP, and ETS, but can promote binding of TFs of the homeodomain, POU, and NFAT class.

We applied the fully automatized multi-omics analysis web service “My Genome Enhancer” (MGE) (my-genome-enhancer.com) to identify DNA methylation patterns that can serve as epigenetic biomarkers. As input to MGE we took the RNA-seq and DNA methylation data previously generated on the tumor samples obtained from the patients of colorectal cancer together with samples of non-affected gut mucosa from the same patients. MGE uses the database on gene regulation TRANSFAC®, the signal transduction pathways database TRANSPATH®, and software that employs a genetic algorithm to reveal some of the DNA methylation positions and potential causative biomarkers of colorectal cancer. 47 of the proposed biomarkers underwent experimental testing on an independent set of blood samples from patients with colorectal cancer. As a result, using advanced methods of statistics and machine learning, a minimum set of 6 biomarkers was selected, which together achieve the best cancer detection potential. The markers include hypermethylated positions in regulatory regions of the following genes: CALCA, ENO1, MYC, PDX1, TCF7, ZNF43.

## Methods

### Samples

In the framework of the SysCol project [5], the samples for the analysis were obtained from biobanks of colorectal tissue at four Surgical Departments, located at three major hospitals, within the Central Denmark Region, were further processed at Aarhus University and prepared for transcriptomics and DNA methylation analysis. Tissues were collected from adenomas and carcinomas together with matched normal mucosa, whenever possible. DNA and RNA was extracted from these samples and was sent to Institut d’Investigació Biomédica de Bellvitge, Spain (IDIBELL) for DNA methylation analysis (using 450 K Affymetrix microarrays) and to Université de Genève, Switzerland for RNA sequencing. In the present study, we used data from altogether 313 tumor samples and 30 normal colon mucosa samples. These data were published in the earlier publications coming from these groups [6, 9].

For testing of obtained biomarkers in this work we used a set of samples from an independent cohort of patients from oncological clinics in Moscow and Novosibirsk, Russia. Subject of this study was a cohort of patients without cancer diseases (patients who had colonoscopy for the diagnosis of inflammatory colon diseases, 100 patients) and patients with colorectal cancer (102). We randomly split these samples into the validation samples of 90 CRC and 88 control samples and the test sample of 12 CRC and 12 controls. Blood samples from all study participants were obtained from City Clinical Hospital №1, Novosibirsk. The detailed cohort information is given in the Additional file 1: Table S1. From each patient, 8 ml of plasma was obtained. Plasma samples (about 4 ml) were prepared by centrifugation at 1000 g for 10 min. Immediately after receiving the plasma samples were frozen and stored at − 20 °C until the test period. The specified volumes and modes of preparation and storage of biological samples are common for a standard diagnostic laboratory.

During the tests, 6 markers were determined in the plasma samples. We measured the number of methylated CpG dinucleotides in the following loci (cg06972019 [ENO1], cg02991571 [PDX1], cg00163372 [MYC], cg01421342 [CALCA], cg24093411 [TCF7], cg02612618 [ZNF43]) in the samples. In short, the procedures for the isolation of DNA from plasma samples; procedures for reverse transcription; procedures for bisulphite modification and amplification of DNA were carried out according to the standard protocols. The pyrosequencing reaction was carried out using the PyroMark Gold Q96 Reagents (Qiagen) reagent kit, using PyroMark Q96 ID (Qiagen) pyrosequencer device according to the manufacturer’s instructions. The percentage of methylated CpG was calculated automatically using the PyroMark Q24 Analysis Software software. All measurements were performed in triplicate.

### Sample preparation protocol

The protocol of preparation of samples from the SysCol study has been published in [6, 9]. A short description of the protocol is given in the Additional file 1: Supplement Methods, Sample preparation protocol.

The protocol of preparation of samples from the independent validation cohort of CRC patients was the following.

Peripheral whole blood samples (202) were collected in a 8.5 mL PPT™ tube (Becton Dickinson) containing a gel barrier to separate the plasma after centrifugation. All samples were processed at room temperature within 2 h from the time of blood extraction. Plasma was separated from the cellular fraction by centrifugation at 1500 g for 10 min at 4 °C. After centrifugation, plasma samples were stored immediately at − 80 °C until cell-free DNA (cfDNA) extraction.

Cell-free DNA was isolated using the QIAamp Circulating Nucleid Acid (QCNA) Kit (QIAgen, Valencia, CA, USA), as specified by the manufacturer.

Bisulfite conversion of cfDNA was performed using the EZ DNA Methylation-Gold Kit. Methylation of 47 CpG-locus was measured by Pyrosequencing. The sequences of PCR and sequencing primers used for each assay are shown in the Additional file 1: Table S2.

Further details of the protocol are described in the Additional file 1: Supplement Methods, Sample preparation protocol.

### Methylation microarray analysis

Genomic DNA (gDNA) was isolated from the samples used for gene expression assay, the interphase and organic phenol-chloroform phase of TRIzol®Reagent (Life Technologies, USA). A total of 1 μg of gDNA was bisulfite converted by the EZ DNA Methylation™ Kit (Zymo Research, USA) according to manufacturer’s protocol. After that, for genome-wide screening of methylation events we used (Illumina Infinium Human Methylation450 BeadChip). This platform interrogates 487,734 CpGs (around 21,000 well-annotated genes). All procedures were performed according to the standard Illumina protocol. Arrays were scanned on the Illumina iScan. Overall chip performance and the quality of the raw data were checked using Illumina GenomeStudio (Methylation Module) software in accordance with the manufacturer’s instructions. The raw intensities data were quantile-normalized. Methylation level of each CpG locus was calculated as methylation beta-value (β = intensity of the methylated allele (M) / (intensity of the unmethylated allele (U) + intensity of the methylated allele (M) + 100).

The level of methylated cytosine was determined in percentage, based on the ratio of the height of peaks T and C in the analysed sequence YG. All calculations were performed automatically using the software PyroMark CpG SW1.0. For each locus, the average values of the methylation level of CpG extracellular plasma DNA were calculated in the group with CRC and in the control group. The reliability of differences in the level of methylation of CpG in the compared groups was determined using the T-test; as a threshold significance level, *p* = 0.05 was used.

### RNA-sequencing

For determining the transcriptional state of CRC tumors, the partners of the SysCol project have sequenced total RNA of 313 tumor and 30 normal colon mucosa samples that were obtained from the CRC patients from the clinic as it was described in the Method section above. The samples were paired-end sequenced with a read length of 49 bp on the Illimina HiSeq platform. We attained a mean read count of approximately 57 million reads, ~ 30 million of which were exonic. After sequencing the we have conducted read mapping on the reference human genome (build hg19) and performed a standard statistical analysis of differentially expressed genes using Limma R package incorporated into the My-Genome-Enhancer tool.

### Statistics

All statistical analysis and basic bioinformatics analysis of DNA methylation and RNA-seq data was done using most up-to-date R packages of Bioconductor [10] and Galaxy [11] integrated in the geneXplain platform [12]. Gene Ontology-term enrichment analysis was performed using geneXplain platform own tool that applies hypergeometric test (*p*-value< 0.01).

### Promoter analysis

To identify master regulators potentially orchestrating the changes of gene expression observed in the course of carcinogenesis, on the first step, we carried out large-scale analyses of enriched motifs in promoters of differentially expressed genes (from − 1000 to + 200). The enrichment analysis was conducted using the F-Match algorithm [13]. F-Match takes information (all known TF motifs) from the TRANSFAC® database [14, 15]. The motifs are specified using position weight matrices (PWMs) that give weights to each nucleotide in each position of the DNA binding motif for a TF or a group of TFs. For each PWM, the algorithm finds a score threshold that gives an optimal overrepresentation of the predicted TF binding sites in the regulatory regions of interest (Yes sequences) compared to a background (No sequences). Through several iterations for each chosen PWM score threshold the algorithm compares the frequency of found motifs in Yes sequences with their background frequency in No sequences. F-Match applies a hypergeometric test and reports those motifs (and corresponding transcription factors) whose frequency in Yes set is significantly higher than in the background No set. The optimal score maximizes the odds for a predicted binding site being located in a Yes sequence while satisfying a chosen statistical significance threshold. The applied implementation optimizes the Yes/No discrimination with respect to two criteria. The first criterion (site overrepresentation) takes into account all predicted binding sites and tests for overrepresentation by the binomial test. The second (sequence overrepresentation) seeks to maximize the enrichment of sequences containing at least one binding site in the Yes set and applies the Fisher test for statistical significance. The false discovery rate is controlled by estimating the adjusted *p*-value (using Benjamini-Hochberg procedure).

### Composite module analyst with correlation analysis (CMAcorrel)

Composite Module Analyst (CMA) builds a model of gene regulatory regions, which consists of one or several composite regulatory modules. The structure of the model and the algorithm applied to build the models is described in detail in our previous reports [16, 17]. Here we present a new variant of the algorithm that constructs the model of gene regulatory regions with the structure correlating with quantitative genomic characteristics, such as gene expression value or value of DNA methylation in particular genomic region or ChIP-seq peak height or any other value that may correlate with the composition of TFBS in the regions under study.

Each composite module (CM) can be represented as duplet (Μ,Ψ), where Μ is a set of positional weight matrices (PWMs) included in the module and Ψ is a set of parameters: an average width of the module (*σ*); number of PWMs (*K*), number of best sites *κ*^(*k*)^ recognized by the PWMs in the sequence that are taken into account by the algorithm, cut-offs ${q}_{cut- off}^{(k)}$ associated with PWMs. All these parameters are not specified at the beginning of the analysis, their values are to be identified by the algorithm itself.

Once the parameters of CM are specified, the CM can be applied to classify any nucleotide sequence. It uses the Match™ algorithm to search for potential TF sites in the sequence *s* under study by applying the PWMs from Μ with the cut-offs ${q}_{cut- off}^{(k)}$. After that, the algorithm selects the *κ*^(*k*)^ of the matches of the PWM *k* with the highest scores (${q}_j^{(k)}>{q}_{cut- off}^{(k)}$) and it goes through all positions *x* in the sequence *s* of the length *l(s)* and finds the position *x*_*max*_ that maximizes the *cm_score*(*s*) according to the the following equation (which is a modified version of the score presented in [16]):

1 $$ cm\_ score(s)=\underset{x=1.l(s)}{\max}\sum \limits_{k=1,{K}_i}{\sum}_{j=1}^{\upkappa^{(k)}}{q}_j^{(k)}(s)\times f\left(\left|x-{\theta}_j^{(k)}(s)\right|,0,{\sigma}^2\right) $$

where ${q}_j^{(k)}(s)$ is the score of *j-*th match of the *k*-th PWM in the sequence *s* and ${q}_j^{(k)}>{q}_{cut- off}^{(k)}$; the *f(x,0,σ* ^*2*^*)-* is the function of normal distribution with mean = 0 and standard deviation *σ;* ${\theta}_j^{(k)}(s)$ - is the position of the *j-*th match of the *k*-th PWM in the sequence *s.*

So, the *cm_score(s)* finds the position *x*_*max*_ in each sequence *s* with the highest concentration of TF binding sites for the given PWMs, since the sites located in the vicinity of the central position *x*_*max*_ contribute more to the score than the sites that are located on a larger distance from this position. The contribution of the sites to the score is regulated by the function of the normal distribution with the standard deviation *σ* which corresponds to the avarage “width” of the composite module.

**Fig. 1:**
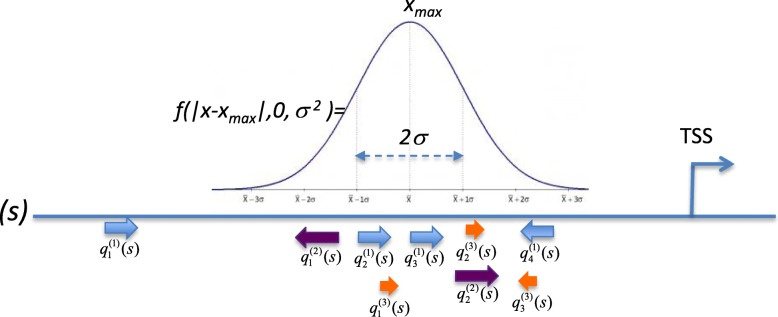
Schematic representation of a match of a composite module (CM) in a particular promoter *(s)* (TSS – transcription start site). Several TF sites are concentrated around position *x*_*max*_ found by the algorithm in the sequence

If the *cm_scores*(*s*) is higher then a predefined threshold *cm_score*_*cut-off*_, the program reports a match of the composite module to the sequence (Fig. 1).

The regulatory region of a gene, for instance, 5′-regulatory region, which is usually considered as a genomic region of several hundred nucleotides upstream of the TSS (start of transcription) plus one or two hundred nucleotides downstream of TSS, may contain sub-regions corresponding to several different CMs. Each such sub-region contains a cluster of sites for several TFs that bind to DNA in synergistic (or antagonistic) manner leading to particular regulation (enhancement or repression) of expression of the target gene. Such sub-regions correspond to specific enhancers or silencers acting on the target genes. So, the full model of the regulatory regions of the genes of our focus contains several CMs and computed additively according to the following equation:

$$ reg\_ score(s)=\sum \limits_{\tau =1}^{\mathrm{T}} cm\_{score}_{\tau }(s) $$

here, *T* is the number of CMs in the full model of the regulatory regions.

The parameters of the full model of the regulatory regions are found by an optimization strategy based on the genetic algorithm. The genetic algorithm uses a fitness function for directing the optimization of the parameters of the model. In our previous work [16] we have defined a fitness function that uses two sets of regulatory regions, a positive set (Yes set) and a negative set (No set), and maximizes the discrimination between these two sets. In the current paper we describe a new fitness function that takes the full set of regulatory regions and actually optimizes the correlation between the computed composite score of the regulatory region and a quantitative value reflecting a functional characteristic of the regulation regions under study, such as the expression value (or expression fold change) of the target genes or the value of DNA methylation in a particular genomic region or ChIP-seq peak height or any other value.

The genetic algorithm proceeds through several iterations of generating “populations” of models, introducing random “mutations” of model parameters, and selecting models characterized by highest values of the fitness function. The output of *CMAcorrel* is the model of regulatory regions under study that gives the best-achieved correlation between the model score and the quantitative value associated with these regulatory regions.

Below we describe the details of the fitness function used in the *CMAcorrel* algorithm.

Let’s have a set of pairs:

$$ P=\left\{\left(s, value\right)|\ s\in S\right\} $$

where *s* are the regulatory sequences from the set *S* of regulatory sequences under study; *value* is the quantitative value functionally associated with sequence *s* (for instance LogFC of the gene expression changes of the genes whose promoters we are analyzing in the given study). First, we search for TF binding sites applying Match™ (using TRANSFAC PWMs) for the whole set *S* of sequences*.* The results of the site search are then forwarded to the *CMAcorrel.*

Let’s define the following set of pairs that is computed for a given model of the regulatory regions:

$$ {P}_{score}=\left\{ reg\_ score(s), value\right)\left|\left(s, value\right)\in P\right\}=\left\{\left({X}_i,{Y}_i\right)\right\}. $$

For the sets of values *X*_*i*_ and *Y*_*i*_ we can compute the ranks ${rg}_{X_i}$ and *rg*_*Y*_ . Let’s define the value of “correlation” as the coefficient of rank correlation of Spearman that is calculated according to the following formula:

$$ correlation=\frac{\mathit{\operatorname{cov}}\left({rg}_X,{rg}_Y\right)}{\sigma_{rg_X}{\sigma}_{rg_Y}}, $$

where *cov*(*rg*_*X*_, *rg*_*Y*_) is the covariance of the rank values, and ${\sigma}_{rg_X},{\sigma}_{rg_Y}$ are the standard deviations of the ranks.

So, the final fitness function for a given model is:

$$ fitness(model)=-{complexity}^{- penalty}\lg \left(1-\left| correlation\right|\right), $$

where *penalty* is a free parameter defined by the user, *complexity* is the complexity of the model, which is usually computed as a total number of different PWMs included into the model.

As the result, *CMAcorrel* returns the best model and also a set of TFs that are associated with the PWMs that were included into the model. The set of TFs is used then for the algorithm of identification of master regulators.

### Identification of master regulators

We define those molecules (or genes) as master regulators that regulate the expression of the differentially expressed genes through concerted control of the activity of those TFs acting on these genes. Master regulators are identified by applying the key-node analysis algorithm published earlier [13], which has been introduced into the Genome Enhancer tool used in this work. The algorithm uses the TRANSPATH® database on gene regulatory and signal transduction pathways [18]. All signal transduction reactions from TRANSPATH® (including ligand binding reactions, phosphorylation and de-phosphorylation reactions, complex formation reactions, ubiquitination and other reactions known from the scientific literature) are considered as a weighted and directed graph. For an input set of TFs the algorithm searches in the graph for potential common regulators (key-nodes) using a modified shortest path algorithm. The key-nodes are then prioritized according to a score that is computed on the basis of the weighted ratio between the number of molecules from the input set that can be reached from the key-node in a limited number of steps (radius parameter) and the total number of reachable nodes in the graph. The higher the score the greater is the chance that the key-node plays the master regulatory role in the process in focus.

### Algorithm of identifying “walking pathways”

We have developed a modification of the master-regulator search algorithm that considers potential rewiring of cancer pathways due to dynamic epigenetic changes such as changes in DNA methylation status of gene regulatory regions during cancer development. We call this method search for “Walking Pathways”, since it attempts to identify rewiring of the signaling pathways at their “legs” – at the most downstream level of the pathways when activated transcription factors are binding to their binding sites in genome. In cases when the TF binding sites appear to be closed at some genomic regions of “normal” tissues and are opened at other genomic regions in “tumorous” tissues due to the epigenetic changes in the genome, the respective TFs are binding to the new places and consequently the most downstream layer of signaling pathways is changing – “the pathway legs are walking”. Such “walking” can lead to the abnormal gene regulation which happens due to the epigenetic rewiring of the target DNA sequences of some signaling pathways.

The search of the walking pathways is done detecting positive feedback loops in the network that is demonstrated in the of the Additional file 1: Fig. S1. We assume that due to TF binding to the epigenetically altered regions of a genome a number of genes are changing their level of expression (which is detected by genome-wide transcriptomics measurements, e.g. using RNA-seq method). In turn, such changes of expression of genes encoding the corresponding TFs as well as other components of the signaling network upstream of these TFs may lead to an increase of the activity of the whole signaling cascade involved in the gene regulation. We hypothesize that the presence of multiple positive feedback loops in the signaling pathway characterizes the most active state which is achieved by the network system during its dynamic rewiring (pathway walking) in the course of carcinogenesis. Therefore, search for the network structure with multiple feedback loops will enable us to identify the active carcinogenic pathways and also to find the master-regulators of these pathways. We will consider various components of such pathways as potential causative biomarkers that will be taken for further experimental verification.

For detection of pathways with positive feedback loops we apply the “Context algorithm” that was described in our previous publication [19]. The algorithm takes into account information of the up-regulated gene products (context information) during the master-regulator search. The context algorithm modifies costs of the edges in the signaling network that are adjacent to the nodes representing products of the up-regulated genes. The idea of the approach is to direct the master-regulator search algorithm (e.g. the underlying Dijkstra algorithm for shortest paths) towards such nodes by decreasing the total costs of the path through these nodes in the network. As a result, the algorithm will search for the network sub-structures that contain one or more feedback loops.

### Pipeline “My-Genome-Enhancer.com”

**Fig. 2:**
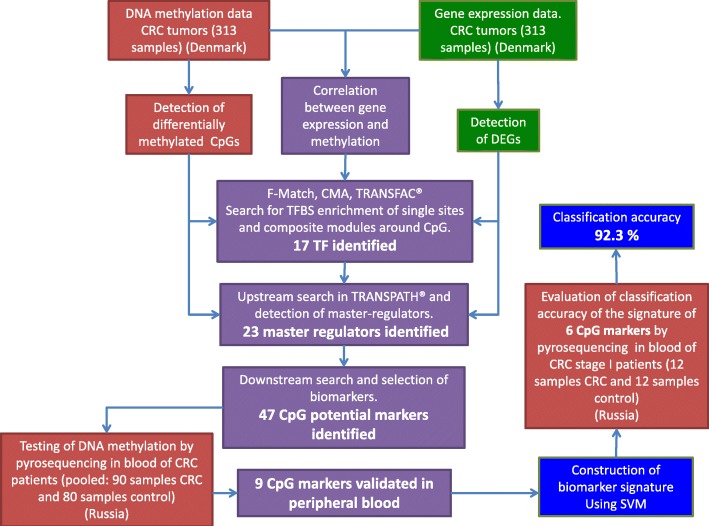
General scheme of the data analysis pipeline applied in this study

The complete analysis of RNA-seq and DNA methylation data was performed with the help of the fully automatized multi-omics analysis web service “My Genome Enhancer” (MGE) (my-genome-enhancer.com). MGE uses the rich environment of bioinformatics software and databases of the geneXplain platform [12]. MGE uses the database on gene regulation TRANSFAC® [14] (release 2017.2), the signal transduction pathways database TRANSPATH® [18] (release 2017.2), and software that employs AI (artificial intelligence) methods such as genetic algorithms for the identification of potential cancer-specific enhancers and employs also advanced methods of graph analysis for identification of master regulators in signal transduction pathways. In Fig. 2 we provide a general scheme of the pipeline of data analysis that was performed in this paper.

All the tools and algorithms described in this paper (including the F-Match, CMA and master-regulator search) are available in the geneXplain platform upon free registration. The results of the analysis obtained in the study are available for the readers in the geneXplain platform in the “Example” section of the platform.

## Results

### Identification of differentially expressed genes

In the first step of the analysis the differentially expressed genes (DEGs) were identified from the gene expression data. We applied the Limma tool (R/Bioconductor package integrated into our pipeline) and compared gene expression of the genes in Cancer samples versus Control samples. Limma calculated the LogFC (the logarithm to the base 2 of the fold change between these different conditions), the *p*-value and the adjusted p-value (corrected for multiple testing) of the observed fold change. As a result we detected 690 upregulated (Additional file 2: Table S3) and 455 downregulated (Additional file 2: Table S3) genes (adjusted *p*-value< 0.05, LogFC> 1.5 for up-regulated and LogFC<− 1.5 for down-regulated).

### Functional classification of target genes

A functional analysis of genes that were differentially expressed was done by mapping the up- and down-regulated genes to several available ontologies, such as Gene Ontology (GO), disease ontology (based on HumanPSD™ database) and the ontology of signal transduction and metabolic pathways from the TRANSPATH® database. Statistical significance was computed using a binomial test. Additional file 3: Figs. S2-S4 show the most significant categories. Among the most significant GO categories we revealed: increased nucleic acid metabolic process; among signaling pathways: S phase and Aurora-A cell cycle regulation; and among diseases: Digestive System Neoplasms. All these results reveal genes that are clearly involved in carcinogenic processes related to the colorectal cancer.

### Comparison of samples with different tumor stages

**Fig. 3:**
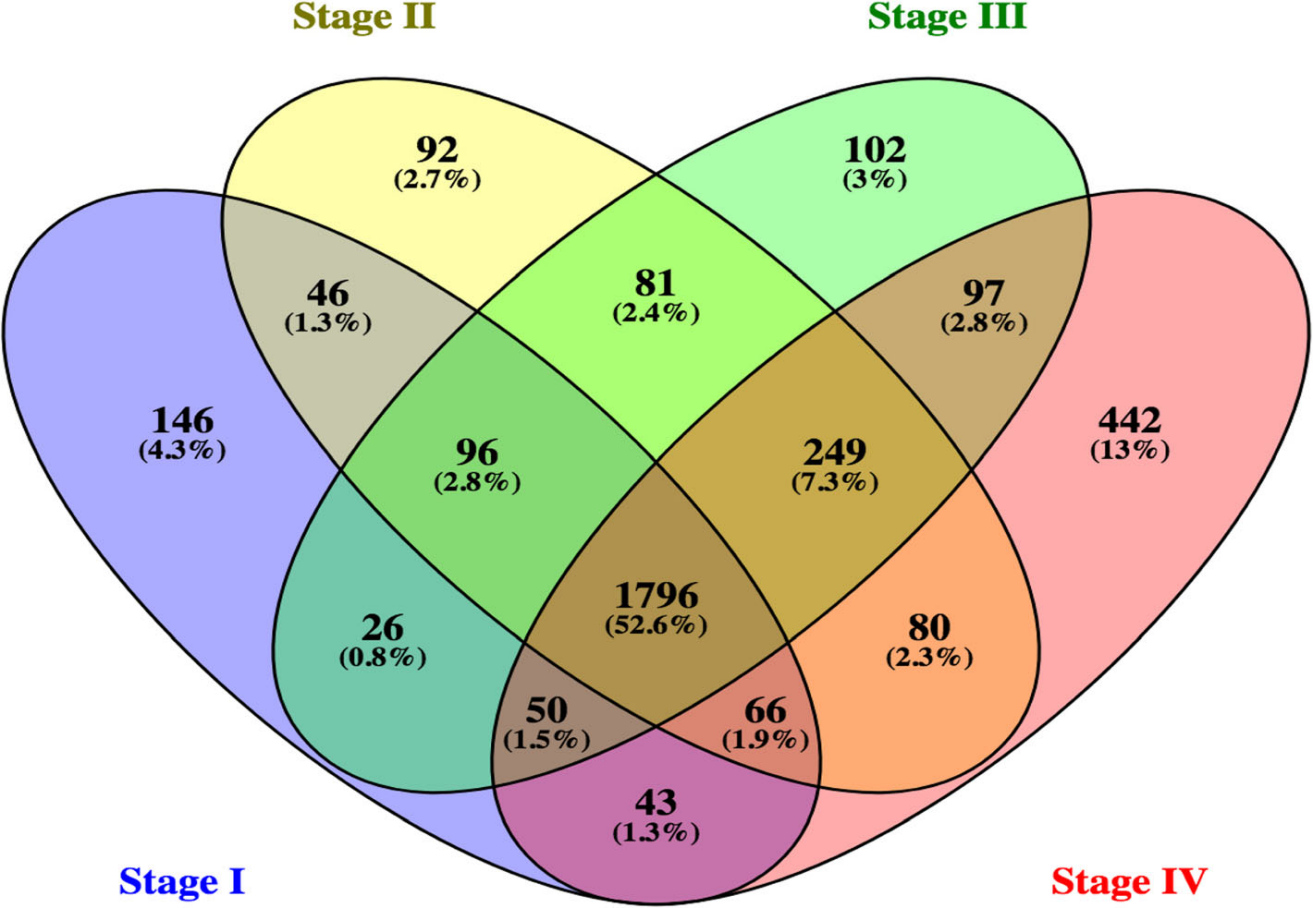
Venn diagram of the number of differentially expressed genes revealed for each tumor stage

Next, we separately analysed samples with different tumor stages. We analysed the following number of samples: 33 adenomas, 44 samples of CRC stage I, 124 samples of CRC stage II, 93 samples of CRC stage III and 19 samples of CRC stage IV. Figure 3 shows Venn diagrams of the differentially expressed genes (DEGs were identified by comparing samples of particular grade with samples of normal tissues with the following filtering parameters: LogFC> 1.0 or < − 1.0 and adj. p-value< 0.05).

We can see that the majority of DEGs, more than 50%, are common to all tumor stages. These common genes belong to the following main GO categories: “regulation of cell migration”, “ion homeostasis”, “regulation of cell communication”, “regulation of cell proliferation”. The second largest group with 13% are the DEGs that are specific for the stage IV, indicating that at the latest cancer stage activates significant additional genetic programs. Analysis of TRANSPATH pathways of the genes of this group revealed enrichment of IL-3 signaling, angiotensin pathway. Among highly enriched GO categories: “regulation of immune response” and “regulation of lymphocyte activation”, which fully agrees with the increased role of immune system at the latest stages of tumor development. Since we were particularly interested in the early onset of CRC, we analysed the group of genes specific for the early stage I. This group has got 146 genes and enriched GO terms are: “developmental process”, “regulation of cellular localization”, “regulation of transport”, “lipid catabolic process”. Among the enriched pathways we can see: “pathway of metabolism of Diacylglycerol (DAG)”.

### Analysis of DNA methylation data

The DNA methylation data were generated from the same samples of tumor and normal gut epithelial tissues as RNA-seq data. The data generated were obtained from the EU-supported SysCol project [5]. DNA methylation data were generated using Illumina methylation arrays described in the Methods section. The analysis of DNA methylation data was performed with the help of the fully automatized multi-omics analysis web service “My Genome Enhancer” (MGE) (my-genome-enhancer.com). As it is described in the Methods section, the methylation level of each CpG locus was calculated as methylation beta-value (β). First of all, we applied Limma and identified CpG loci with beta-values significantly higher or lower in tumor samples compared to the samples of normal tissues. Out of 485,513 CpG sites assayed at the Illumina array we identified 25,864 CpGs with beta-value fold change higher 1.5 or lower 0.67 (which is 1/1.5) with adjusted *p*-value < 0.0001. We consider these loci as most interesting for our further study, since the statistically significant difference in DNA methylation values is observed in tumor samples in comparison to samples from the normal tissue.

### Analysis of correlation between DNA methylation and gene expression

Next, we combined the analysis of gene expression (RNA-seq) with DNA methylation data of genomic CpG loci. We analysed correlation (Spearman rank correlation) between beta-values of DNA methylation at each of the 25,864 CpGs selected at the previous step and expression values of genes computed using RNA-seq data. In total, we identified 5746 CpG loci with correlation coefficient higher then 0.18 (critical value of correlation coefficient for p-value < 0.05) with expression of at least one gene in genome. These loci were in our focus of the next analysis steps in the study.

We also searched for CpG methylation sites that are located inside genes and in proximal regulatory regions (+/− 2 kb upstream and downstream) of genes differentially expressed in the tumors of stage-I and with high correlation of methylation level and expression of these genes in different samples. Analysis of the earliest stages of tumor, such as stage-I, was in our focus since our primary goal was to find biomarkers for early detection of CRC. In the Additional file 3: Fig. S5 we give the screenshot from the results of the correlation analysis in MGE showing CpGs loci in the gene loci with the highest correlation coefficient (r > 0.4 or r < − 0.4) between the of DNA methylation of these CpGs and expression of the genes in the stage-I tumor samples. One can see that the correlated CpG sites are often located in 5′-regions, introns or other regulatory regions of these genes. In total, we found 449 CpG loci the methylation level of which negatively correlated with gene expression (NEG – see Additional file 2: Table S5, correlation coefficient < − 0.4) and 339 CpG loci with methylation level positively correlated with gene expression (POS – see Additional file 2: Table S6, correlation coefficient > 0.4).

### Identification of enriched TF binding sites around CpG loci

In order to identify transcription factors that may be activated during initiation and progression of CRC we analysed genomic regions that potentially regulate expression of genes in CRC of various grades. It is known that regulation of gene expression is controlled not only through promoter sequences but also through enhancers and silencers that can be localized in distal upstream regions as well in introns and in downstream regions of genes. In order to identify most probable enhancers and silencers acting in CRC we chose to study genomic regions at CpG loci that demonstrate strong correlation between the level of DNA methylation and the level of expression of genes in their vicinity (located in proximal 5′ and 3′ regions of the genes, in their introns as well as in relatively distal regions, i.e. +/− 2 kb upstream and downstream of the transcribed region). As it was demonstrated recently DNA methylation can influence binding of various transcription factors to their target sites overlapping the methylated regions [20]. In turn, through protein-protein interactions such perturbed binding of one transcription factor can influence binding of other TFs to their binding sites in close vicinity (for references, see TRANSCompel database of TF composite elements) [14]. Such changes, if they happen at an enhancer or silencer of a particular gene, can influence the function of these regulatory regions and consequently alter the gene regulation. In our analysis we took regions of 200 bp around the methylated CpG markers correlated with gene expression in order to reveal potential enrichment of binding sites for transcription factors. In the cases when two or more CpG loci were overlapping within 200 bp regions we combined the overlapping regions.

We applied the F-Match algorithm, which computes the frequency of TF binding sites in the target sequences (YES set) and compares it with the frequency of TFBS in the control sequences (NO set). As control sequences we chose 200 bp regions around the CpG loci in the genome that demonstrate no significant DNA methylation in all our samples. F-Match finds those PWMs and corresponding transcription factors whose sites are statistically significantly overrepresented in the YES set compared to the NO set (see Method section). The results of this analysis are presented in the Additional file 2: Table S7, and Additional file 2: Table S8 (for NEG and POS). Among the most overrepresented TFBS were sites for the factors NF1, HNF3, PAX9, E2F6, SOX9, GR and PDX1..

As it was mentioned above, it is important to understand the interactions between transcription factors during their binding to specific enhancer or silencer region in genome. We have therefore also applied the CMA algorithm (Composite Module Analyst) [16] for searching composite modules to the regions around CpG loci whose methylation level is strongly correlated with the level of gene expression. In the current work we applied both, the classical CMA algorithm as well as the novel modification of the algorithm called CMAcorrel (see Method section) that is able to reveal compositions of TF binding sites that correlate with the level of DNA hyper- or hypomethylation in the target regions. First, we applied CMA and as input we used the same NEG and POS sets of 449 and 339 CpG loci respectively that are characterized by high correlation coefficients (r < − 0.4 and r > 0.4) of methylation and gene expression levels. As above we took regions of 200 bp around CpG sites. The algorithm identified models consisting of two 10 PWMs each. In the Additional file 3: Figs. S6 and S7, we present a screenshot from the geneXplain platform with detailed information about composite modules that were found in the methylated regions of our interest and also the statistical parameters of the constructed model.

**Fig. 4:**
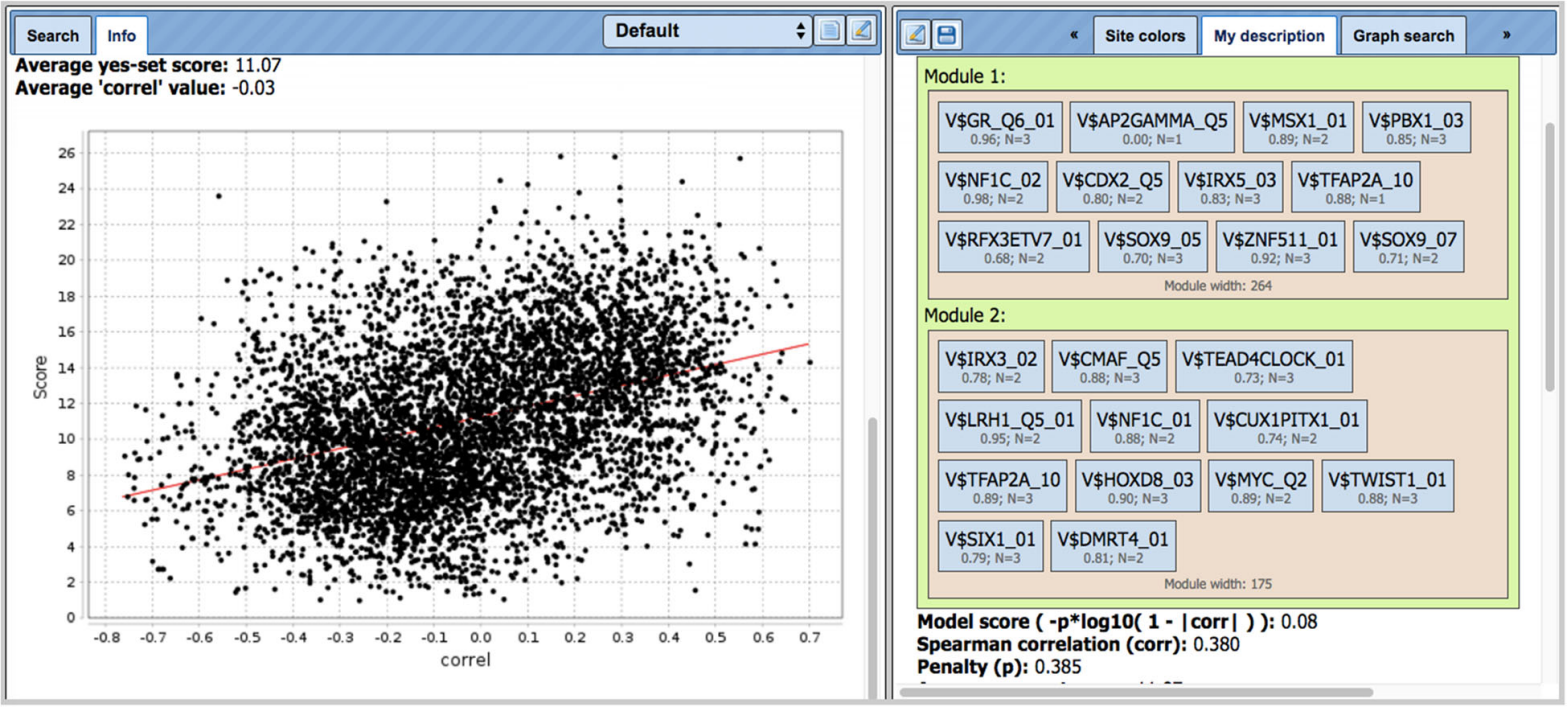
A screenshot of the results of CMAcorrel for analysis of 5746 CpG loci with correlation coefficients higher then 0.18 (and r < − 0.18) between the levels of DNA methylation and gene expression. We analysed 500 bp regions around each CpG. At the right there is the composite model consisting of two composite modules with 10 PWMs each. At the left there is the plot of DNA-methylation-gene expression correlation versus the composite score of the region around CpG. Spearman correlation coefficient = 0.38. PWMs are the Position Weight Matrices (PWMs) selected by CMAcorrel algorithm to be included into the model consisting of two composite modules. Below each matrix name are the cut-off values given that were optimized by the CMAcorrel algorithm (in cases of cut-off = 0.0 the original profile cut-off was chosen by the algorithm). The parameter N (e.g. *N* = 2) gives the number of top scoring TF sites in the sequence that were considered for score calculation. The module width is the sigma value of the score (see Methods section)

Additionally, we applied the novel algorithm CMAcorrel to the full initial set of 5746 CpG loci with correlation coefficients higher then 0.18 (and r < − 0.18). Moreover, we took a larger area around CpG sites of 500 bp, in order to check the bigger context of the DNA methylation sites. The results are shown in Fig. 4.

Finally, we combined TFs identified by classical CMA analysis (of NEG and POS sets versus control), by CMAcorrel as well as by F-Match of enriched single PWMs. We obtained 71 genes encoding TFs linked to the obtained PWMs in the results of all these enrichment analyses (see, Additional file 4: Table S9). After further filtering these TFs according to their differential expression as well as differential DNA methylation and the level of correlation with the methylation in the associated CpG loci we came up with the list of 19 TFs that we used for further analysis (Table 1).

**Table 1:** The final list of 19 TFs after filtering according to their differential expression as well as differential DNA methylation and the level of correlation with the methylation in the associated CpG loci

Ensembl	TF gene symbol	TF protein name	correl	logFC I_stage vs. Control	logFC Cancer vs. Control	Methylation logFC Cancer vs. Control	CMA NEG_200bp	CMA POS_200bp	CMAcorrel_500bp	F-Match NEG_200bp	F-Match POS_200bp
ENSG00000198521	*ZNF43*	ZNF43	−0.763	− 0.706	− 0.711	0.296	+				
ENSG00000257591	*ZNF625*	ZNF625	−0.667	−0.461	− 0.365	0.558	+				
ENSG00000136997	*MYC*	c-Myc	−0.567	2.126	2.442	−0.682			+		
ENSG00000197905	*TEAD4*	TEF-3	−0.453	1.829	2.335	−0.589			+		+
ENSG00000139515	*PDX1*	IPF-1	−0.407	2.565	2.869	0.541				+	+
ENSG00000125798	*FOXA2*	HNF-3beta	−0.364	0.821	1.312	−0.332	+	+			
ENSG00000177426	*TGIF1*	TGIF	−0.282	1.003	1.511	−0.294	+				+
ENSG00000204103	*MAFB*	MafB	−0.244	−0.678	− 0.226	0.280		+			
ENSG00000149948	*HMGA2*	HMGI-C	−0.242	0.533	1.016	−0.284		+			+
ENSG00000137309	*HMGA1*	HMGIY	−0.220	1.422	1.758	−0.328	+	+			
ENSG00000169016	*E2F6*	E2F-6	−0.204	0.793	1.259	−0.420	+				
ENSG00000196628	*TCF4*	ITF-2	−0.166	−0.429	0.021^a^	0.332		+			
ENSG00000159216	*RUNX1*	AML1	−0.057	0.911	1.525	−0.556	+				
ENSG00000075426	*FOSL2*	Fra-2	−0.050	−0.559	− 0.027^a^	−0.386	+			+	
ENSG00000164749	*HNF4G*	HNF-4gamma	0.064	−0.708	− 0.209	− 0.365	+	+			
ENSG00000129514	*FOXA1*	HNF-3alpha	0.183	−1.296	−0.768	− 0.430	+	+			
ENSG00000156127	*BATF*	B-ATF	0.222	0.607	1.028	−0.416		+			
ENSG00000176842	*IRX5*	IRX2a	0.355	0.890	1.155	0.400		+	+	+	
ENSG00000113580	*NR3C1*	GR	0.548	−1.331	−0.931	− 0.588	+	+	+		

^a^ The LogFC was not significant for the full Cancer vs Control comparison, but was highly significant for the Cancer stage I vs Control

### Search for master-regulators and reconstruction of networks with positive feedback loops

On the next step of the analysis we applied the master-regulator search algorithm that searches in the network upstream of transcription factors found in the previous step. As it is described in the Methods section, we applied the strategy of “walking pathways” when the upstream search algorithm is bound to search for the paths through the signal transduction network that are associated with multiple positive feedback loops that may cause the carcinogenic aberrations in gene expression. MGE started the search with the set of 19 TFs reveled at the previous step (see Table 1) and has run the master-regulator search through the TRANSPATH® database with the maximum number of 10 upstream steps with the “Context Analysis” option using all up-regulated gene products as “context molecules” in the algorithm. We used all genes that demonstrated significant up-regulation (LogFC> 1.0 adj.*p*-value < 0.05) at least in one of the above-mentioned comparisons of tumor samples with control (all tumor samples versus all normal samples, stage specific tumor samples versus normal samples and metastatic samples compared to non-metastatic). As a result we obtained a list of 273 potential master-regulators fitting to all these criteria representing 97 genes (several isoforms of the same protein were considered as independent potential master-regulators by the algorithm) (see, Additional file 5: Table S10). Further prioritization of this list according to the level of differential gene expression in all cancer samples and particularly in the I stage of cancer stages and also according to the level of the differential DNA methylation in cancer versus control sets led us to select the final list of 23 genes as most important master-regulators (Table 2) in the system.

**Table 2:** Selected 23 genes as potential master-regulators prioritized according to the level of differential gene expression in different cancer stages and in metastatic cancer and also according to the level of the differential DNA methylation in cancer versus control sets

Master molecule name	Gene symbol	Meth probes: Illumina ID	correl	logFC I_stage vs. Normal	Cancer_vs_Normal logFC	Meth_logFC	Number of target TFs^a^	Master-regulator Score
MKP-2	*DUSP4*	cg13635007	− 0.018	1.694	2.309	−0.408	13	0.751
c-Myc	*MYC*	cg00163372	−0.498	2.126	2.442	−0.682	13	0.725
IL-17A	*IL17A*	cg11924517	−0.145	1.083	0.889	−0.747	13	0.649
MT1-MMP	*MMP14*	cg05931439	−0.212	0.793	1.439	−0.486	13	0.619
eNOS	*NOS3*	cg08018731	−0.059	1.460	1.958	−0.631	13	0.560
TGFbeta-2A	*TGFB2*	cg06899755	−0.171	0.645	0.946	0.296	13	0.556
IGF-2	*IGF2*	cg02425416	−0.025	0.760	1.353	−0.419	13	0.517
col1A1	*COL1A1*	cg18618815	−0.142	1.340	2.085	−0.428	13	0.502
Matrin	*MMP7*	cg01813071	−0.055	4.505	4.814	−0.367	13	0.498
CTLA-4	*CTLA4*	cg08460026	−0.110	0.892	1.022	−0.699	13	0.496
amphiregulin-NTF	*AREG*	cg02334660	−0.438	1.649	1.711	−0.644	13	0.494
alpha-enolase	*ENO1*	cg06972019	−0.405	0.783	1.148	−0.653	13	0.480
CXCR2	*CXCR2*	cg06547715	0.005	1.156	1.036	−0.570	13	0.479
calcitonin	*CALCA*	cg01421342	0.052	0.834	1.043	0.309	13	0.455
IRAK-2	*IRAK2*	cg09386682	−0.419	1.292	1.614	−0.444	13	0.446
WT1	*WT1*	cg01952234	−0.227	0.412	1.004	0.346	13	0.415
IL-11	*IL11*	cg26367719	0.082	1.789	2.039	−0.568	13	0.401
Wnt-2	*WNT2*	cg07697895	0.128	2.171	2.494	0.288	13	0.385
CD86^b^	*CD86*	cg00697440	0.141	−0.153	0.105	−0.584	13	0.384
GROalpha	*CXCL1*	cg00419314	−0.145	3.922	3.903	−0.313	11	0.378
trip6	*TRIP6*	cg00374672	−0.292	0.949	1.356	−0.610	11	0.363
mgat5	*MGAT5*	cg20063095	−0.209	0.570	1.162	−0.459	13	0.343
Fcgamma RIIIB	*FCGR3B*	cg04567009	0.048	1.709	1.727	−0.573	11	0.284

^a^ 13 target TFs: AML1a, E2F-6, Fra-2, GR-alpha, HMGI-C, HMGIY, HNF-3beta, HNF-4gamma, ITF-2-A-, SEF2-1A, TGIF, c-Myc, IPF-1; 11 target TFs: AML1a, E2F-6, Fra-2, GR-alpha, HMGI-C, HMGIY, HNF-3beta, HNF-4gamma, TGIF, c-Myc, IPF-1

^b^ CD89 did not achieve statistical significant levels of gene expression Fold Changes but was selected here due to its highly significant level of methylation Fold Change

**Fig. 5:**
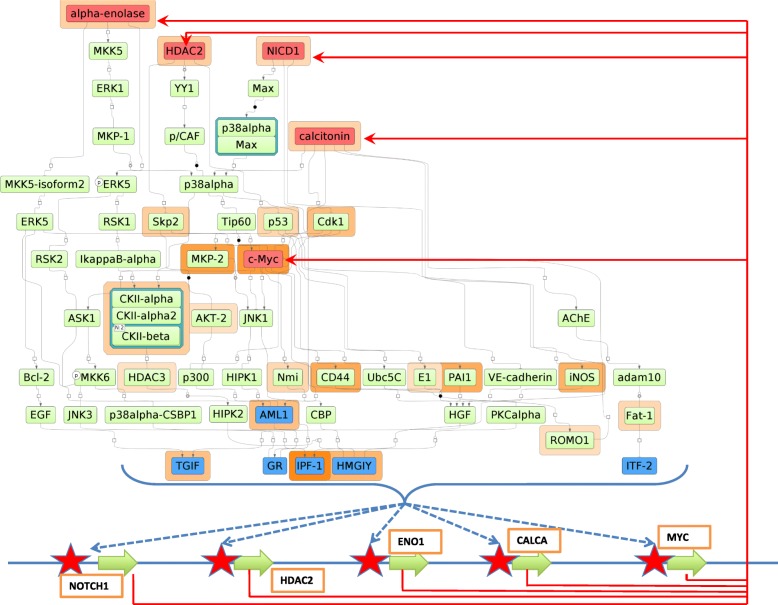
A fragment of a diagram of the signal transduction network combined with the gene regulatory network predicted by MGE workflow as playing a regulatory role in CRC. Red nodes represent master-regulators identified by the network analysis algorithm. Blue nodes represent transcription factors predicted by CMA in the gene regulatory regions of the differently expressed genes (green arrows on a blue lines at the bottom). Red stars represent methylated CpG loci identified in our work whose methylation level correlates with expression level of the genes. Red arrows show translation of the genes into proteins making the multiple feedback loops in the system. The products of the differentially expressed genes play the master-regulatory role in the system. Brown and violet shading around some nodes in the network shows the level of up-regulation or down-regulation of the genes encoding these proteins

In Fig. 5 we show a fragment of a diagram of the signal transduction network combined with the gene regulatory network constructed by the workflow of My-Genome-Enhancer. This network is predicted as playing one of the key regulatory roles in different subtypes of CRC. Red nodes represent master-regulators identified by the network analysis algorithm. Blue nodes represent transcription factors predicted by CMA in the gene regulatory regions of the differentially expressed genes. The genes are represented as green arrows on a blue line at the bottom of the diagram. Red stars represent methylated CpG loci identified in our work whose methylation level correlates with the expression level of the genes. This network helps us to identify the causative DNA methylation biomarkers for early detection of CRC.

### Selection of a target list of potential biomarkers

Taking into account all selected TFs and the connected upstream master-regulators we looked downstream for the identified binding sites of these TFs in the CpG loci under study and compiled a list of related CpG loci as potential biomarkers, which will be subject for further prioritization and experimental validation. Such upstream search followed by downstream TFBS lookup identifies those particular CpG loci that are under the direct control of the identified master-regulators.

As the result of the procedure described above we have selected the following set of 47 DNA methylation biomarkers (Table 3).

**Table 3:** Selected set of 47 potential DNA methylation biomarkers

ID	CHR	Position (hg19)	Gene symbol (gene)	Methylation Caner_vs_Control LogFC	Expression Cancer_vs_Control logFC	Correlation	Enriched TF^a^	Master-regulator^b^
cg02612618	19	22,018,605	*ZNF43*	0.296	−0.711	−0.743	+	
cg07945582	7	26,206,579	*NFE2L3*	−0.534	2.858	−0.518	+	
cg00163372	8	128,752,988	*MYC*	−0.682	2.442	−0.498	+	+
cg02915837	12	3,069,243	*TEAD4*	−0.306	2.335	−0.453	+	
cg02334660	4	75,312,483	*AREG*	−0.644	1.711	−0.438		+
cg09386682	3	10,207,069	*IRAK2*	−0.444	1.614	−0.419		+
cg06972019	1	8,937,448	*ENO1*	−0.653	1.148	−0.405		+
cg01777575	20	22,566,140	*FOXA2*	−0.332	1.312	−0.307	+	
cg19377250	7	100,463,206	*TRIP6*	−0.712	1.356	−0.292		+
cg01952234	11	32,457,130	*WT1*	0.346	1.004	−0.227		+
cg05931439	14	23,305,957	*MMP14*	−0.486	1.439	−0.212		+
cg20063095	2	134,977,141	*MGAT5*	−0.459	1.162	−0.209		+
cg17726575	2	11,606,945	*E2F6*	−0.420	1.259	−0.204	+	
cg18696576	6	34,203,630	*HMGA1*	−0.328	1.758	−0.190	+	
cg06899755	1	218,520,325	*TGFB2*	0.296	0.946	−0.171		+
cg01742897	18	53,257,019	*TCF4*	0.184	0.021	−0.166	+	
cg15555970	18	3,452,317	*TGIF1*	−0.294	1.511	−0.161	+	
cg00419314	4	74,735,092	*CXCL1*	−0.313	3.903	−0.145		+
cg11924517	6	52,050,597	*IL17A*	−0.747	0.889	−0.145		+
cg18618815	17	48,275,324	*COL1A1*	−0.428	2.085	−0.142		+
cg08460026	2	204,732,474	*CTLA4*	−0.699	1.022	−0.110		+
cg00425708	12	66,217,779	*HMGA2*	−0.284	1.016	−0.105	+	
cg08018731	7	150,687,961	*NOS3*	−0.631	1.958	−0.059		+
cg01813071	11	102,401,616	*MMP7*	−0.367	4.814	−0.055		+
cg02425416	11	2,163,808	*IGF2*	−0.419	1.353	−0.025		+
cg13635007	8	29,210,154	*DUSP4*	−0.408	2.309	−0.018		+
cg07330438	21	37,258,460	*RUNX1*	−0.556	1.525	−0.011	+	
cg06547715	2	218,990,976	*CXCR2*	−0.570	1.036	0.005		+
cg08836542	2	28,618,831	*FOSL2*	−0.386	−0.027	0.006	+	
cg02059626	8	76,319,264	*HNF4G*	−0.365	−0.209	0.026	+	
cg04567009	1	161,600,769	*FCGR3B*	−0.573	1.727	0.048		+
cg01421342	11	14,995,754	*CALCA*	0.309	1.043	0.052		+
cg26367719	19	55,875,605	*IL11*	−0.568	2.039	0.082		+
cg01830294	7	116,963,492	*WNT2*	0.132	2.494	0.128		+
cg01664670	20	39,316,308	*MAFB*	0.280	−0.226	0.140	+	
cg01824511	14	38,064,456	*FOXA1*	−0.430	−0.768	0.141	+	
cg00697440	3	121,795,768	*CD86*	−0.584	0.105	0.141		+
cg01589587	14	76,002,440	*BATF*	−0.416	1.028	0.222	+	
cg24093411	5	133,449,651	*TCF7*	0.387	2.022	0.321	+	
cg02991571	13	28,501,126	*PDX1*	0.541	2.869	0.353	+	
cg06613263	5	142,779,552	*NR3C1*	−0.588	−0.931	0.410	+	
cg03130910	1	234,908,226	*BMP3*	−1.107	−2.482	0.601		
cg05259836	6	74,290,516	*PYY*	−0.813	−5.604	0.601		
cg24032190	15	67,442,893	*ADH1B*	−1.071	−3.781	0.603		
cg04786142	1	234,908,381	*NR5A2*	−1.075	−1.644	0.606		
cg03800922	6	74,290,220	*CA1*	−0.842	−6.934	0.610		
cg26541218	7	47,826,387	*KLF4*	−0.771	−1.938	0.617		

^a^The column “Enriched TF” marks genes that encode TFs whose sites found enriched around CpG loci

^b^The column “Master regulator” marks genes that encode master-regulator molecules identified in the study

During next steps of biomarker prioritization we performed comparison of correlation between pairs of markers. It is clear that we need in order to select the least correlated markers in order and to achieve the highest classification power with the a minimal number of markers. We computed the correlation coefficient in for each pair of markers using the DNA methylation values obtained in tumor as well as in control samples. For instance, a high correlation of markers in the MYC and AREG genes (corr = 0.876) allowed us to exclude the marker in the AREG gene from the set of potential markers for further study, whereas relatively low correlation of markers in the MYC and CALCA genes (corr = 0.308) prompted us to include both of these markers in the final list of markers.

Hierarchical clustering of markers using the matrix of pairwise correlations between them allowed us to reveal clusters of mutually correlated markers. (Additional file 6: Table S11).

**Fig. 6:**
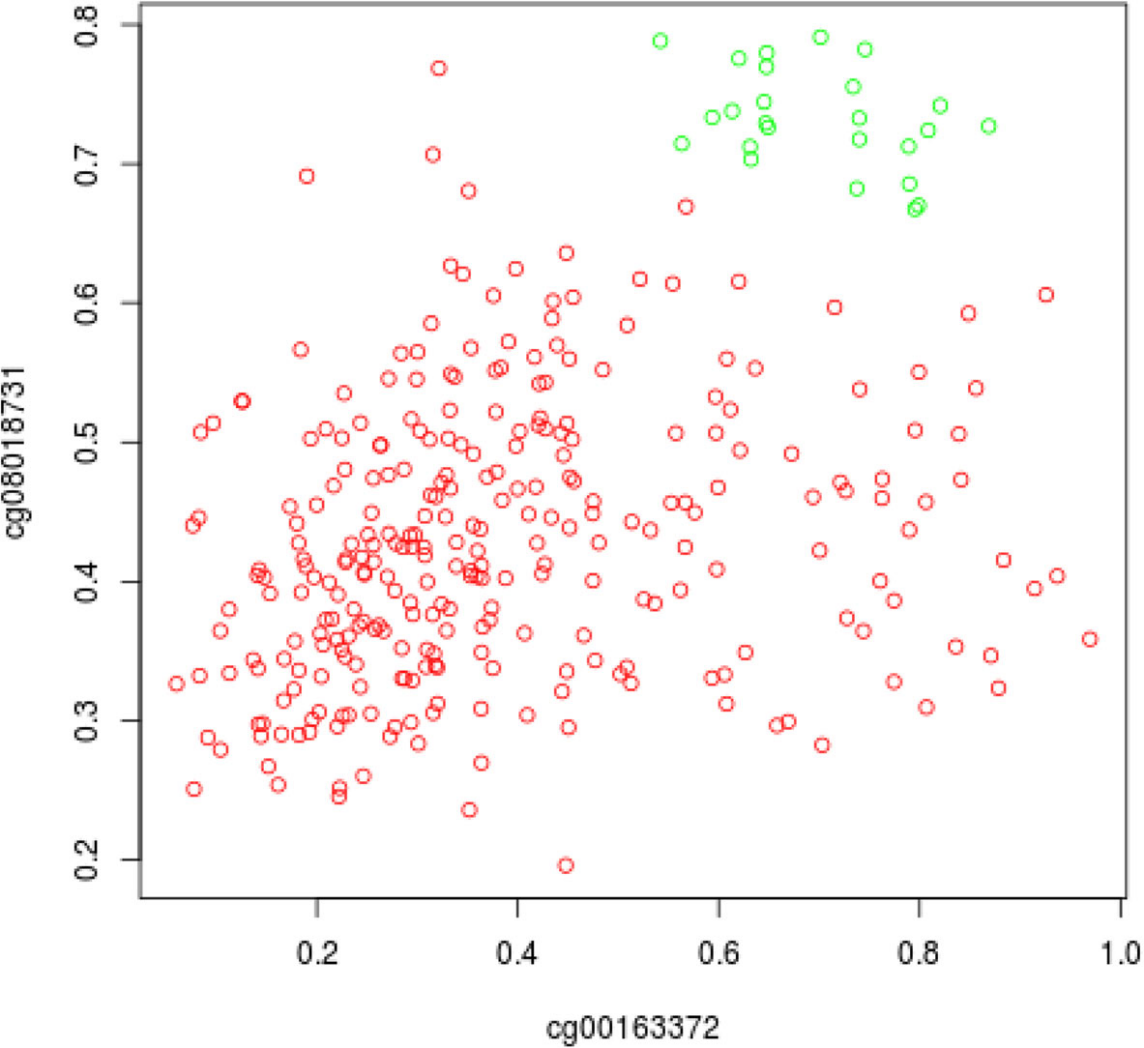
Diagram of DNA methylation values of two markers cg00163372 (in gene *MYC*) and cg08018731 (in gene *NOS3*). The red dots show values obtained in tumor samples, the green dots show values for the normal samples

It was interesting to see that several biomarkers from the list demonstrate high combinatorial potential, so combination of such biomarkers can have a very high predictive power. In the Fig. 6 we demonstrate such potential by comparing the DNA methylation values of two biomarkers from the list in gene *MYC* (cg00163372) and in gene *NOS3* (cg08018731) (nitric oxide synthase 3 (endothelial cell)). One can see a clear separation of the samples obtained from tumor (red) and normal samples (green). This confirms a possible functional connection between these two biomarkers and it demonstrates the advantage of combining several biomarkers together to achieve better classification power.

We feel that such combinatorial potential of our biomarkers will play an important role for testing in clinical samples.

### Testing of the CpG-markers in independent clinical samples

In order to validate the selected 47 CpG biomarkers we tested the level of methylation of these markers in peripheral blood of independent cohort of CRC patients (case) as well as in blood samples of patients without diagnosed CRC (control - patients who had colonoscopy for the diagnosis of inflammatory colon diseases). Testing was done with the help of pyrosequencing of cell-free DNA from blood samples after bisulfite conversion of DNA to differentiate and detect unmethylated versus methylated cytosines in CpG pairs.

So, the testing of the differentially methylated CpGs in clinical samples was done according to the following protocol: (1) extraction of cell-free DNA; (2) bisulfite conversion of DNA; (3) amplification of the tested region by PCR; (4) pyrosequencing and identification of the sequence of the PCR products. The cell-free DNA was extracted from the frozen blood samples as it is described in the Method section.

For all selected 47 CpG loci we obtained the methylation level in ctDNA of peripheral blood of patients from the validation sample diagnosed with CRC (90 samples) and the control group of patients without cancer (88 samples). As it is described in the Method section we randomly divided the case samples and control samples into 6 subgroups of samples of equal sizes and pooled the DNA in each subgroup. For each locus we computed the average level of DNA methylation of the CpG position in the group of CRC patients and in the control group and also computed the standard deviation between pooled subgroups. The statistical significance of the difference of methylation levels between case and control was computed by t-test with *p*-value cut-off *p* < 0.05.

As a result, for 9 loci we confirmed statistically significant differences between CRC patients and cancer-free control patients. The identified CpG loci that are located in the regulatory regions of the following genes: *ENO1, IGF2, CALCA, PDX1, ZNF43, FOSL2, TCF7, DUSP4, MYC.* It is interesting that in the locus *ENO1*, we identified a second CpG near the one that was tested (cg06972019, the CG dinucleotide №3 in the sequence). Both CpG showed significant difference in methylation between cancer and control, but the new CpG showed even higher significance than the initially identified one (CG dinucleotide №2 in the sequence). A similar situation was observed for the CpG locus 29 (*TCF7*): here the testing CpG dinucleotide was №2 (cg24093411), whereas the other one (№1 in the sequence) showed a higher significance in the comparison of cancer to control. Finally, for the locus 37 (DUSP4) the testing CpG did not show any statistical difference, but we found a different CpG located nearby (№1 in the sequence) that showed a statistically significant increase in methylation in the cancer group in the comparison to the control group. The summary of all results of testing is shown in Additional file 3: Table S12.

We use these 9 loci for further selection of perspective biomarkers and creation of the combined signature for CRC diagnostics.

### Creating a diagnostic combination of biomarkers

Finally, selected biomarkers were used to build a minimal combination of biomarkers with a high diagnostic potential. We used the support vector machine (SVM) method to achieve such a goal. SVM was implemented in the geneXplain platform by integrating the R library e1071 [7**]. The SVM method builds a classification function on the basis of an input of the DNA methylation values of all markers measured on a training set of samples. Once the classification function is ready it can be used to classify any new samples. For each sample it computes a classification score, and depending on the level of the critical value of the score the sample is classified then as a tumor or non-tumor sample.

Here we used SVM tools also for selecting the best minimal combination of biomarkers. We started from 9 biomarkers and gradually decreased their number to 6 still keeping reasonably high level of classification accuracy.

We used a set of 12 blood samples from patients of stage I of CRC and 12 blood samples of patients without any recorded oncological disease. Evaluation of the classification accuracy of the marker combinations was done by the method of random division of the set into training and control sub-sets. Each time, we were splitting our set of samples into 50% of training sub-set of samples and 50% of samples into the control sub-set. This random splitting was done 100 times. Each time the parameters of SVM were fitted on the test sub-set and the obtained classification function was tested on the test sub-set. The number of misclassifications was computed and the splitting was done again. After 100 of such random iterations we computed an average accuracy of the classification procedure.

Finally, with the set of 6 CpG markers shown in the Table 4 we were able to construct the classification function that achieved the maximum average value of the classification accuracy of 92.3% in the random permutation tests described above.

**Table 4:** Six DNA methylation markers selected for building CRC diagnostic classification function using SVM method

Probe ID	Chromosome	Gene Symbol	Gene Name
cg01421342	11	CALCA	calcitonin-related polypeptide alpha
cg06972019 (CpG №3)	1	ENO1	enolase 1, (alpha)
cg00163372	8	MYC	v-myc avian myelocytomatosis viral oncogene homolog
cg02991571	13	PDX1	pancreatic and duodenal homeobox 1
cg24093411	5	TCF7	transcription factor 7 (T-cell specific, HMG-box)
cg02612618	19	ZNF43	zinc finger protein 43

More detailed information about these genes (containing chosen DNA methylation markers) and their known role in molecular pathways and diseases (retrieved from HumanPSD database) is given in the Additional file 7: Table S13. We can see that these genes play various roles in different but functionally connected processes, pathways and diseases, which gives a particular strength to these sets of biomarkers.

In the Additional file 3: Fig. S8, we show the web interface of the respective diagnostic tool that is constructed on the basis of the SVM classification model using the combination of 6 selected biomarkers. User inputs DNA methylation values of 6 biomarkers for one or more samples and the tool makes a classification of the samples as CRC or non-CRC. It is accessible at the following URL: [21].

## Discussion

In this paper we have applied a novel approach of detecting causative biomarkers using a strategy that we called analysis of “walking” pathways. This strategy is using our previously developed approach of “upstream analysis” [19, 22] to multi-omics data. We introduced an important extension to the upstream analysis algorithm by searching for positive feedback loops, which directs the search in the gene regulatory and signal transduction network towards potential master-regulators of a self-inducing pathological state of the system. In the current paper, we applied this new strategy to the massive transcriptomics (RNA-seq) and epigenomics (DNA methylation) data of a cohort of 300 patients with CRC. All these experimental data for the biomarker identification were taken from previously published work of the SysCol consortium [8, 9]. An important novel part of the approach is the application of the search for enriched single TFBS and their combinations (composite modules) to genomic regions around differentially methylated CpG loci in early stages of CRC. TFs revealed by this analysis are used then as input in the network search algorithm that is performed with the option of “Context Algorithm” [19], where signal transduction proteins and their complexes encoded by genes up-regulated in CRC tumor samples are used as the “context” nodes. This allows the revelation of potential positive feedback loops in the system, when particular signaling proteins (receptors, their ligands, adaptor proteins, kinases etc.) may exert a positive regulation of their own genes through signal transduction network by activating multiple TFs that in turn bind to their target sites in regulatory regions of those genes and up-regulate them. We call these signaling proteins “master-regulators” of the system. They are often characterized by increased expression and changed DNA methylation pattern in pathological states such as a tumor. Revealing such master-regulators as well as the components of the signaling cascade and consequently activated TFs may help to come up with a good set of biomarkers for the pathological state. We think that the key component in such a discovery is the search for small regions of DNA that get their epigenetic status changed (e.g. through DNA methylation or de-methylation of CpG loci), leading to the altered pattern of TF binding in gene regulatory regions throughout the genome. Our approach gives us the possibility to integrate transcriptomics and epigenomics data in the search for promising causative biomarkers. We applied it to CRC and were able to identify a set of 47 potential DNA methylation biomarkers. We were interested to find such biomarkers that can be detected in DNA circulating in the blood samples of early stages of CRC. We have performed a thorough validation of the proposed CpG loci in the blood samples of an independent cohort of 90 CRC patients comparing the level of the DNA methylation in these 47 loci to 88 of other patients with no detected cancer. Finally, we selected six DNA methylation biomarkers and by applying a robust machine learning technique (SVM) we created a prognostic score computed as a combination of six biomarkers, which achieved sensitivity and specificity levels above 92% (computed on a relatively small sub-set of samples - 12 tumor samples and 12 controls). A considerable part of the biomarker discovery has been done with the help of the automatic pipeline “My-Genome-Enhancer” (my-genome-enhancer.com) of the geneXplain platform. Therefore it can be easily reproduced and can be applied to perform a search for causative biomarkers for other types of cancer.

One potential limitation of the approach described here comes from our still rather simple methods of finding potential TFBS in DNA sequences. Although we use the most comprehensive and up-to-date database, TRANSFAC®, the site search method is based on PWMs, which are quite simple models for TF binding motifs. It has been reported recently [20, 23] that using more advanced models, based on the application of Markov models and being able to take into account possible interactions between nucleotides in the motif, better results in TF site recognition may be achieved in comparison to applying simple PWMs. Still the reported improvement is not very high - only about 36%. Also, it is still not possible to build such more complex models for the majority of known TF. On the other hand, the PWM approach with available data can cover almost all known TFs, which was very important for our approach where a comprehensive coverage of TF motifs plays an important role in the analysis of the specific TF site combinations.

Another important limitation is related to the fact that our knowledge about TFs and their motifs as well as about signal transduction network functioning in the cancer cells is not complete. Thus the networks reconstructed by our algorithms may miss important components that are not known so far. Still, we think that the number of known interactions in the network and known TF motifs is big enough to get a quite robust prediction of the most important master-regulators in the system and the components of their regulatory sub-network. Anyway, the final proof of these predictions must come from experimental validation, which was performed in our work and reported here. Indeed, as we predicted, some of the revealed biomarkers were confirmed in an independent, though still quite small, cohort of CRC patients.

In the recent years, a number of methylated DNA loci that distinguish between healthy and tumor tissues have been identified [24], however, only a few of them have been accepted as blood-based biomarkers for clinical testing and have been integrated into CRC screening [24, 25]. One of the most successful DNA methylation biomarker, SEPT9 has achieved an accuracy of CRC detection in serum samples at 72% [26]. These studies demonstrate that blood-based DNA tests have reasonable predictive power and can be used in clinical screening procedures. Still the accuracy of such methods based on single DNA methylation locus (like SEPT9) is rather limited, especially when applied to an independent and highly heterogeneous cohort of the patients. Mechanism-based selection of biomarker combinations like the one proposed in our study offers a solution for increasing accuracy of the CRC detection since it integrates signals from several biomarkers reflecting potential heterogeneity of various aberrations in cancer pathways. Continued effort is still needed to optimize further the biomarker combinations that can be in future incorporated into clinically available blood tests to identify patients who are precancerous.

## Conclusions

The proposed approach of the search for “Walking pathways” is indeed promising for biomarker discovery applications. In this paper, we analysed an extensive collection of full genome gene-expression data (RNA-seq) and DNA methylation data of genomic CpG islands of about 300 patients with colorectal cancer and identified six potential epigenetic biomarkers of DNA methylation using the approach of searching for “walking pathways” that takes into account positive feedback loops – self-activating circuits that, according to our modeling, may lead to rapid tumor development. The selected markers include hypermethylated positions in regulatory regions of the genes encoding transcription factors as well as other components of signaling pathways: CALCA, ENO1, MYC, PDX1, TCF7, ZNF43. The revealed biomarkers were experimentally validated in an independent, though still quite small, cohort of CRC patients. This confirms the robustness of the revealed biomarkers, which we expected because of their potential causative role in the molecular mechanisms of the considered pathological processes during early stages of development of CRC.

## Additional files

Additional file 1: The MS Office file (.docx format) that gives additional explanations of the methods and contains **Table S1**, **Table S2** and **Figure S1**. (DOCX 113 kb)

Additional file 2 The MS Excel file that contains **Tables S3**-**S8**. (XLSX 507 kb)

Additional file 3: An MS Office file (.docx format) that contains **Table S12** and **Figures S2–S8**. (DOCX 1437 kb)

Additional file 4: **Table S9.** List of 71 genes encoding TFs linked to PWMs that were found overrepresented in the genomic regions around CpGs whose methylation was negatively of positively correlated with gene expression. These TF genes were identified independently by three independent algorithms: by classical CMA analysis (of NEG and POS sets versus control), by CMAcorrel as well as by F-Match of enriched single PWMs. (XLSX 76 kb)

Additional file 5: **Table S10.** The list of 273 potential master-regulators (representing 97 genes - several isoforms of the same protein were considered as independent potential master-regulators by the algorithm) that may control activity of 19 TFs. (XLSX 101 kb)

Additional file 6: **Table S11.** Table of pairwise Pearson correlations between CpG methylation values of pre-selected markers. (XLSX 63 kb)

Additional file 7: **Table S13.** Detailed information about genes that contain chosen DNA methylation markers. We present information about their known role in molecular pathways and diseases (retrieved from HumanPSD database). These genes play various roles in different but functionally connected processes, pathways and diseases related to cancer. (XLSX 32 kb)

## Abbreviations

cfDNA: Cell-free DNA
CMA: Composite module analyst
CRC: Colorectal cancer
ctDNA: Circulating tumor DNA
PWM: Position weight matrix
TF: Transcription factor
TFBS: Transcription factor binding site
