# Supplementary material for: Walking pathways with positive feedback loops reveal DNA methylation biomarkers of colorectal cancer
Source: BMC Bioinformatics. 2019 Apr 18;20(Suppl 4):119. doi: 10.1186/s12859-019-2687-7 (PMC6471696; doi:10.1186/s12859-019-2687-7)
Supplement: Supplementary file 1 — The MS Office file (.docx format) that gives additional explanations of the methods and contains Table S1, Table S2 and Figure S1. (DOCX 113 kb) [file 12859_2019_2687_MOESM1_ESM.docx]

**Supplement Methods**

Table SM1. Information about blood samples from all study participants that were obtained from City Clinical Hospital №1, Novosibirsk. Subject of this study was a cohort of patients with colorectal cancer (102).

| **CRC stage** | **Gender** | **Age** | **Number of blood samples** |
| --- | --- | --- | --- |
| 2-nd stage | male | 54.3±16.4 | 13 |
|  | female | 62.1 ± 11.2 | 8 |
| 3-nd stage | male | 59.3±14.2 | 49 |
|  | female | 58.9 ± 14.0 | 32 |

And a cohort of patients without cancer diseases (patients who had colonoscopy for the diagnosis of inflammatory colon diseases, 100).

| **Disease** | **Gender** | **Age** | **Number of blood samples** |
| --- | --- | --- | --- |
| Irritable bowel syndrome (IBS) | male | 36,5 ± 19,3 | 10 |
|  | female | 38,4 ± 10,8 | 16 |
| Non-specific ulcerative colitis | male | 26,5 ± 8,3 | 4 |
|  | female | 42,2 ± 11,7 | 20 |
| Diverticular disease | male | 52,5 ± 9,3 | 14 |
|  | female | 56,4 ± 6,8 | 20 |
| Crohn's disease | male | 31,5 ± 6,3 | 4 |
|  | female | 28,4 ± 4,8 | 4 |
| No colon pathology | male | 36,5 ± 19,3 |  |
|  | female | 42,4 ± 26,9 | 8 |

Table SM2. The sequences of PCR and sequencing primers used for Pyrosequencing.

| **CpG locus ID** | **Gene whose expression correlate with the DNA methylation at CpG locus** | **Structures of forward (F), reverse (R) and sequencing (Seq) primers** |
| --- | --- | --- |
| cg06972019 | ENO1 | F_5’-gattttaggtgattttgaggaggttta-3’  R_5’-biotin-ccaaacaactaaaaaaactataaataatcc-3’  Seq_5’-ggattataggygtgagttat-3’ |
| cg04567009 | FCGR3B | F_5’-ggtagtttaatttatagttatagatgtggtga-3’  R_5’-biotin-catcaaacaccccttttcaaac-3’  Seq_5’-atttttttgtgggagttttatt-3’ |
| cg06899755 | TGFB2 | F_5’-atagtattagggatttgttttaggaga-3’  R_5’-biotin-tataaacctccttaacrtaatactcttc-3’  Seq_5’-gatttgttttaggagaagg-3’ |
| cg02425416 | IGF2 | F_5’-gggtaatgttgaatgtttttagagg-3’  R_5’-biotin-atcacactaaacccttttcctaatc-3’  Seq_5’-ttagagggtttggagggta-3’ |
| cg01421342 | CALCA | F_5’-gtttaatttttaagatgtttttaattgattt-3’  R_5’-biotin-cccaaccccraattattattcc-3’  Seq_5’-gatttttttttttttaattatag-3’ |
| cg01952234 | WT1 | F_5’-gtttaatttttaagatgtttttaattgattt-3’  R_5’- biotin-aacctcctaactcctcctcttc-3’  Seq_5’-aagtttaaataagaggggt-3’ |
| cg01813071 | MMP7 | F_5’-gagtttatagaattttgaaagtatgtgtta-3’  R_5’-biotin-cctaccaataacratataatacttcctc-3’  Seq_5’-gaaagtatgtgttattttttat-3’ |
| cg00425708 | HMGA2 | F_5’-gtagtttagaygttttttgtaaagtgttg-3’  R_5’-biotin-ccacrtttcraaacttaacctttacctac-3’  Seq_5’-gttttttgtaaagtgttggtt-3’ |
| cg02991571 | PDX1 | F_5’-ggattattttgtttttttttgtagga-3’  R_5’-biotin-aaaaaccrtcraatacaccacc-3’  Seq_5’-tgtaggaagtttttagatgg-3’ |
| cg05931439 | MMP14 | F_5’-ggtttaggaatttaagtttagtgtttat-3’  R_5’-biotin-ccraacccacrccctaaaatc-3’  Seq_5’-gtgtttatygaagataaagg-3’ |
| cg01824511 | FOXA1 | F_5’-gagygttttygygggaagtgag-3’  R_5’-biotin-ccacatcaactcaactacacttacct-3’  Seq_5’-tgagygggttgtttttg-3’ |
| cg01589587 | BATF | F_5’-gagaatattgtagggataggatagatagagt-3’  R_5’-biotin-tttccaacctaacttccaactaacta-3’  Seq_5’-gataggatagatagagtttttgatt-3’ |
| cg18618815 | COL1A1 | F_5’-gaaggagtatgaatttgtatagagagtgt-3’  R_5’-biotin-ccaaaatccccctaataaacct-3’  Seq_5’-tgtttattgaagttttaggtt-3’ |
| cg15555970 | TGIF1 | F_5’-gttggggattaaggttgggtt-3’  R_5’- biotin-aaccctatacrccctaaaataaaaac-3’  Seq_5’-gtatyggtgggaattt-3’ |
| cg19597776 | TCF4 | F_5’- ggattatagagaaaagagaaattattgagaa-3’  R_5’- biotin- ctcatctttaatttcttaataactac-3’  Seq_5’- tatttttttygtatatttatatt-3’ |
| cg02612618 | ZNF43 | F_5’-gatttttttgtggtttttgtataatttgg-3’  R_5’-biotin-cctacaaccctctataaacaactctaca-3’  Seq_5’-ttgtataatttgggagaga-3’ |
| cg17726575 | E2F6 | F_5’-gttagtgttgatgtttagtaaatgtgagta-3’  R_5’-biotin-caaacaaacraacaaacaaaaataaaac-3’  Seq_5’-gagtaattatattattgtggaat-3’ |
| cg08836542 | FOSL2 | F_5’-aaggattgattttagaggtttttgtt-3’  R_5’-biotin-ctaatatataataaaattaccttaaacccaa-3’  Seq_5’-agtttatgttatgattttgtt-3’ |
| cg20063095 | MGAT5 | F_5’-ggatagtatagttgtgtgaattttaaattg-3’  R_5’-biotin-ccataccaaccttataactttactataacact-3’  Seq_5’-aattgttttgaggtgttt-3’ |
| cg08460026 | CTLA4 | F_5’- ggtttgttttgtttagttgagtgtttg-3’  R_5’-biotin- tacacatatacacacacaaaaaacacttaa-3’  Seq_5’- gaygtaatagttaaattta-3’ |
| cg06547715 | CXCR2 | F_5’-ggattttttttttgtaatttaggttagaagt-3’  R_5’-biotin-cctcaaaattaaataataatcaaaactattaaaca-3’  Seq_5’-gtaatttaggttagaagttttat-3’ |
| cg01777575 | FOXA2 | F_5’-ggttgggaggttgagatttgt-3’  R_5’-biotin-cctcrattataactaaaaaataaccttaaaacac-3’  Seq_5’-gatttgtttttgatatttaggt-3’ |
| cg01664670 | MAFB | F_5’-ttyggttttgttygagtttagga-3’  R_5’-biotin-acaaaaaaccttaactaaaacraaaataac-3’  Seq_5’-ttgttygagtttaggagg-3’ |
| cg07330438 | RUNX1 | F_5’-gtatttaagtgattaaggttattgttatttgaa-3’  R_5’-biotin-ctaaataatatttaaaatccaaatatcctaactc-3’  Seq_5’-gatttagtagtgatttgaagt-3’ |
| cg09386682 | IRAK2 | F_5’-ttaggtggttttttatttagtttataatagtt-3’  R_5’-biotin-gattttgtagggatttayggtgttg -3’  Seq_5’-gtttataatagtttgtaaagtg-3’ |
| cg00697440 | CD86 | F_5’-ggtttagggtttagtggttaagttag-3’  R_5’-biotin-ctcatccacacaaaaacrtataatacaa-3’  Seq_5’-gttaagttaggattggattt-3’ |
| cg00419314 | CXCL1 | F_5’-ggttttttagttttaattatgtataaaagg-3’  R_5’-biotin-cctacracccraactctataactctc-3’  Seq_5’- attatgtataaaaggggtt-3’ |
| cg02334660 | AREG | F_5’-ggagtygattatgattatttagaagagtatg-3’  R_5’-biotin-ccctactcacctctaactaaatcatc-3’  Seq_5’-aatatttggttatattgt-3’ |
| cg24093411 | TCF7 | F_5’-gagttttygggttgtaggttttag-3’  R_5’-biotin-accratcctccaaatcccaa-3’  Seq_5’-gggttgtaggttttagtta-3’ |
| cg06613263 | NR3C1 | F_5’-ttgtttaattattttaggggtgtaga-3’  R_5’-biotin-caaaccccaataatataacactacc-3’  Seq_5’-attttaggggtgtagagtt-3’ |
| cg18696576 | HMGA1 | F_5’-ttttgtattataggaggtgtggaa-3’  R_5’-biotin-caaaccracccraaaccttct-3’  Seq_5’-ggaggtgtggaaggt-3’ |
| cg11924517 | IL17A | F_5’- gttgtttggtagtatgtagggttggaatatg-3’  R_5’-biotin- taacaattcttttattaaatatctactctactcaa-3’  Seq_5’- atatgtttttaatagaaaattt-3’ |
| cg07945582 | NFE2L3 | F_5’-agatgaataaatgaaaggtttagttaaag-3’  R_5’-biotin-aatttacccctaaaaataccct-3’  Seq_5’-gaaaggtttagttaaagtagt-3’ |
| cg00374672 | TRIP6 | F_5’- ggtatgaagtttaataygttggttttaggt-3’  R_5’-biotin- ctaccctccctaatactatctacaaactc-3’  Seq_5’- atygtaggattattttttga-3’ |
| cg07697895 | WNT2 | F_5’- gttgataaagttttaaaygatgggtttag-3’  R_5’-biotin- ctacrcctaaaaaattaaactataaac-3’  Seq_5’- gtttagygagygataaaggttagtt-3’ |
| cg08018731 | NOS3 | F_5’-gttgtttttttattggttagggttg-3’  R_5’-biotin-ctcttacccctactctctttaaaataac-3’  Seq_5’-ggttagggttggattatat-3’ |
| cg13635007 | DUSP4 | F_5’-gaggttgttyggtggtaggat-3’  R_5’-biotin-acractttaaccactaatctccca-3’  Seq_5’-ggtggtaggattttgg-3’ |
| cg02059626 | HNF4G | F_5’-ttagaggtttaaggtgtagtttga-3’  R_5’-biotin-acaactctaaaaccacaccactaa-3’  Seq_5’-taaggtgtagtttgaagaa-3’ |
| cg00163372 | MYC | F_5’-ggatatygaggagaatgttaagagg-3’  R_5’-biotin-ctaatcacrcaaaacaaaaaaactc-3’  Seq_5’-gagygttagaggaggaa-3’ |
| cg03130910 | BMP3 | F_5’-gtattgtatggttgagggtggaa-3’  R_5’-biotin-tatctatcataaaaaaatatcatcaaaaac-3’  Seq_5’-aaataatatttgaaataggt-3’ |
| сg04786142 | NR5A2 | F_5’-ttaaattttgaggtaatgagaagtt-3’  R_5’-biotin-ctaaaaccacactaaaaaaaaccct-3’  Seq_5’-gtaatgagaagtttttagatg-3’ |
| сg24032190 | ADH1B | F_5’-gtgtagtttgtttataggaagtgatttatg-3’  R_5’-biotin-ccrcctcctacaatcaaatctac-3’  Seq_5’-gaagtgatttatggtgttt-3’ |
| сg03800922 | CA1 | F_5’-ggttttgaggttttagtgtgatt-3’  R_5’-biotin-aaaaaaaatttaaaataactcaaccc-3’  Seq_5’-tagtgtgattttttattgtt-3’ |
| сg05259836 | PYY | F_5’-gagtggtttaaatttggaggatt-3’  R_5’-biotin-cttaaaatacattacaaattcactacccta-3’  Seq_5’-aggatttttttggtgtt-3’ |
| сg26541218 | KLF4 | F_5’-gttygagtaaggagtagggtaaga-3’  R_5’-biotin-aaactactactaattaaacaaaaaaacctc-3’  Seq_5’-aaggagtagggtaagagtt-3’ |
| сg10059167 | LINC00395 | F_5’-ttttatgtggttattttggaatg-3’  R_5’-biotin-caccaatacacaatcaataaatatacc-3’  Seq_5’-ggttattttggaatgttta-3’ |

**Sample preparation protocol:**

The protocol of preparation of samples from the SysCol study (published in [4,5]) was the following.

Initially hematoxylin and eosin (H&E) stained tissues were cut from each biopsy to enable histological assessment. This assessment among other things includes an estimation of the fraction of cancer cells in each tumor biopsy (a minimum 60% is required). If necessary, Microdissection was applied to enrich the fraction of cancer cells to the required level. Subsequently, RNA and DNA extraction was performed, each from 25 tissue sections of 10 μm thickness. After sectioning of 25 sections a H&E section was made for evaluation of the cancer cell percentage – to ensure that only if all sections cut from the biopsy contained >60% cancer cells, the RNA and DNA were taken on for RNA-seq and DNA methylation analysis. Finally, the quantity of RNA and DNA was assessed by Qubit measurement. RNA quality was assessed by Agilent Bioanalyzer analysis, with the requirement being a RNA Integrity Number (RIN) >5.4. DNA quality was estimated by gel electrophoresis – requirement: high molecular weight DNA (>200 Kb, with minimal smear from degraded DNA).

The standards for sample collection and preparation are in accordance with the policies and standards provided by the ICGC consortium (E.6 Quality Standards of Samples; http://www.icgc.org/icgc/goals-structure-policies-guidelines/e6-quality-standards-of-samples). This ensures that our sample and data generated from them provide a robust and consistent resource and allow comparisons between tumour types at a later stage.

Processed samples were shipped for DNA methylation analysis and RNA sequencing only if both the tumor and the matched normal mucosa fulfilled the following requirements:

• RNA with RIN >5.4, quantity >2 μg

• DNA with high molecular weight, quantify > 2 μg

The protocol of preparation of samples from the independent validation cohort of CRC patients was the following.

Peripheral whole blood samples (202) were collected in a 8.5 mL PPT™ tube (Becton Dickinson) containing a gel barrier to separate the plasma after centrifugation. All samples were processed at room temperature within 2 h from the time of blood extraction. Plasma was separated from the cellular fraction by centrifugation at 1,500 g for 10 min at 4 ℃. After centrifugation, plasma samples were stored immediately at −80 ℃ until cell-free DNA (cfDNA) extraction.

Cell-free DNA was isolated using the QIAamp Circulating Nucleid Acid (QCNA) Kit (QIAgen, Valencia, CA, USA), as specified by the manufacturer.

Bisulfite conversion of cfDNA was performed using the EZ DNA Methylation-Gold Kit. Methylation of 46 CpG-locus was measured by Pyrosequencing. The sequences of PCR and sequencing primers used for each assay are shown in the Supplementary material Table SM2. Amplicons were generated in a 40µl reaction volume with 15pmol of forward and reverse PCR primers, 0.2mM dNTPs, 1.5mM MgCl2, 1X Buffer II (Applied Biosystems), 1U AmpliTaq Gold (Applied Biosystems) using 0.5-1ng genomic DNA. PCR conditions were 94°C for 7 min; 50 cycles with denaturation at 94°C for 30s, annealing at 55°C for 30s and elongation at 72°C for 30s; 1 cycle at 72°C for 7 min; and a final hold at 4°C. Single-stranded biotinylated PCR products were prepared for sequencing using the Pyrosequencing™ Vacuum Prep Tool. 3µl Streptavidin Sepharose HP (Amersham) was added to 37µl Binding buffer (10 mM Tris-HCl pH 7.6, 2M NaCl, 1 mM EDTA, 0.1% Tween 20) and mixed with 40µl PCR product for 10 min at room temperature using a Variomag Monoshaker (Camlab). The beads containing the immobilised templates were washed with 70% ethanol for 5 sec, denaturation solution (0.2M NaOH) for 5 sec and washing buffer (10 mM Tris-Acetate pH 7.6) for 5 sec. After washing the beads were released into a PSQ 96 Plate Low containing 45µl annealing buffer (20 mM Tris-Acetate, 2 mM MgAc2 pH 7.6), 0.3µM sequencing primer. Primer annealing was performed by incubating the samples at 80 °C for 2 minutes and allowed to cool for 5 minutes at room temperature. Pyrosequencing reactions were performed according to the manufacturer’s instructions using the PSQ 96 SQA Reagent kit (Biotage AB). The sample methylation was determined using the PyroMark CpG Software SW1.0 (Biotage AB).


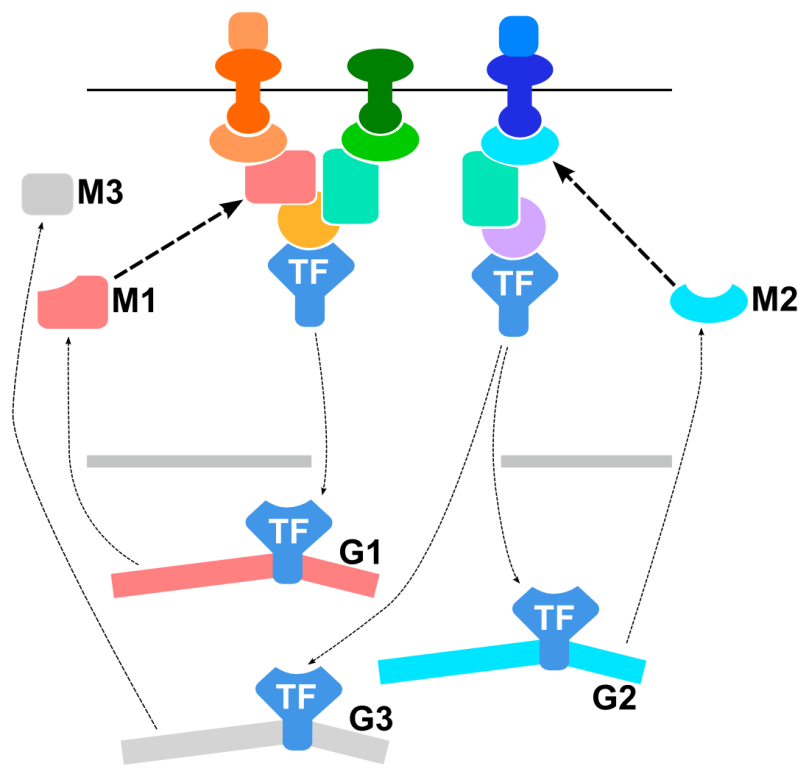


Figure SM1. Schema of feedback loops in the signaling network rewired during carcinogenesis. The genes G1 – G3 are controlled by transcription factor TF. G1 and G2 encode signaling molecules that play a role in the cascades that regulate TF’s activity, so that their regulation by TF represents a positive feedback loop to the affected network cascades.
