# Supplementary material for: Walking pathways with positive feedback loops reveal DNA methylation biomarkers of colorectal cancer
Source: BMC Bioinformatics. 2019 Apr 18;20(Suppl 4):119. doi: 10.1186/s12859-019-2687-7 (PMC6471696; doi:10.1186/s12859-019-2687-7)

**Supplement Results**

**Tables.**

**Supplement Table SM12**

Results of testing of CpG methylation biomarkers on independent samples of ctDNA from peripheral blood of CRC patients compared to control group of patients.

| № | Gene that contains the DNA methylation site in the regulatory region | CpG code of the locus | Level of DNA methylation in control group (%)  ± st.dev. | Level of DNA methylation in the CRC group (%)  ± st.dev. | P< 0.05 |
| --- | --- | --- | --- | --- | --- |
| 1  2 | *ENO1* | cg06972019 (CG №3) | 45.5±7.1 | 20.3±3.1 | P<0.05 |
|  |  | (CG №2) | 86.2±12.1 | 19.3±4.2 | P<0.05 |
| 3 | *FCGR3B* | cg04567009 | 34.2±2.34 | 24.9±0.3 |  |
| 4 | *TGFB2* | cg06899755 | 45.6±4.45 | 58.4±3.4 |  |
| 5 | *IGF2* | cg02425416 | 6.3±2.4 | 25.1±4.9 | P<0.05 |
| 6 | *CALCA* | cg01421342 | 5.6±4.4 | 19.1±8.9 | P<0.05 |
| 7 | *WT1* | cg01952234 | 60.5±9.6 | 51.2±9.6 |  |
| 8 | *MMP7* | cg01813071 | 13.4±2.3 | 10.8±0.7 |  |
| 9 | *HMGA2* | cg00425708 | 9.4±4.5 | 5.6±3.7 |  |
| 10 | *PDX1* | cg02991571 | 6.2±4.5 | 33.3±11.2 | P<0.05 |
| 11 | *MMP14* | cg05931439 | 25.3±9.3 | 16.7±9.9 |  |
| 12 | *FOXA1* | cg01824511 | 48.2±4.48 | 62.7±5.4 |  |
| 13 | *BATF* | cg01589587 | 21.9±4.8 | 30.6±5.5 |  |
| 14 | *COL1A1* | cg18618815 | 35.3±9.6 | 27.4±10.8 |  |
| 15 | *TGIF1* | cg15555970 | 61.3±9.6 | 70.4±10.6 |  |
| 16 | *TCF4* | cg19597776 | ND(*) | ND |  |
| 17 | *ZNF43* | cg02612618 | 8.8±3.5 | 18.5±3.3 | P<0.05 |
| 18 | *E2F6* | cg17726575 | 35.6±6.35 | 26.6±7.3 |  |
| 19 | *FOSL2* | cg08836542 | 7.9±2.4 | 19.8±4.4 | P<0.05 |
| 20 | *MGAT5* | cg20063095 | 42.8±9.6 | 34.7±7.2 |  |
| 21 | *CTLA4* | cg08460026 | ND | ND |  |
| 22 | *CXCR2* | cg06547715 | 35.5±4.3 | 35.7±3.3 |  |
| 23 | *FOXA2* | cg01777575 | 41.7±8.4 | 47.7±7.4 |  |
| 24 | *MAFB* | cg01664670 | 8.1±6.4 | 12.2±7.3 |  |
| 25 | *RUNX1* | cg07330438 | 27.8±2.2 | 34.3±4.2 |  |
| 26 | *IRAK2* | cg09386682 | 27.9±9.2 | 30.8±10.2 |  |
| 27 | *CD86* | cg00697440 | 59.8±3.1 | 53.1±3.2 |  |
| 28 | *CXCL1* | cg00419314 | 47.1±9.4 | 52.4±10.4 |  |

| 29 | *AREG* | cg02334660 | 33.4±8.3 | 27.4±6.3 |  |
| --- | --- | --- | --- | --- | --- |
| 30 | *TCF7* | cg24093411 | 12.8±6.4 | 29.9±3.7 | P<0.05 |
|  |  | (CG №1) | 6.8±5.4 | 45.1 | P<0.05 |
| 31 | *NR3C1* | cg06613263 | 43.2±4.2 | 51.7±3.5 |  |
| 32 | *HMGA1* | cg18696576 | 63.1±3.6 | 81.4±1.6 |  |
| 33 | *IL17A* | cg11924517 | ND | ND |  |
| 34 | *NFE2L3* | cg07945582 | 43.6±2.4 | 43.7±3.4 |  |
| 35 | *TRIP6* | cg00374672 | ND | ND |  |
| 36 | *WNT2* | cg07697895 | ND | ND |  |
| 37 | *NOS3* | cg08018731 | 23.2±8.2 | 19.6±7.8 |  |
| 38 | *DUSP4* | cg13635007 | 40.9±2.5 | 29.6±4.3 |  |
|  |  | (CG №1) | 8.0±3.5 | 18.1±4.1 | P<0.05 |
| 39 | *HNF4G* | cg02059626 | 20.8±9.1 | 14.1±10.7 |  |
| 40 | *MYC* | cg00163372 | 85.2±6.9 | 46.1±15.4 | P<0.05 |
| 41 | *BMP3* | cg03130910 | 62.3±8.5 | 54.8±9.9 |  |
| 42 | *NR5A2* | сg04786142 | 30.6±6.5 | 36.1±8.2 |  |
| 43 | *ADH1B* | сg24032190 | 64.4±4.9 | 68.6±6.4 |  |
| 44 | *CA1* | сg03800922 | 15.6±3.7 | 13.9±3.5 |  |
| 45 | *PYY* | сg05259836 | 15.9±5.8 | 17.9±3.5 |  |
| 46 | *KLF4* | сg26541218 | 56.7±1.2 | 57.9±2.6 |  |
| 47 | *LINC00395* | сg10059167 | 37.4±8.1 | 32.9±6.2 |  |

(*) ND- not detected. We failed to detect the level of methylation of these loci due to a problematic structure of the primers.

**Figures.**

**Supplement Figure SM2**

Enriched GO (biological process) of upregulated genes in Cancer vs. Control.


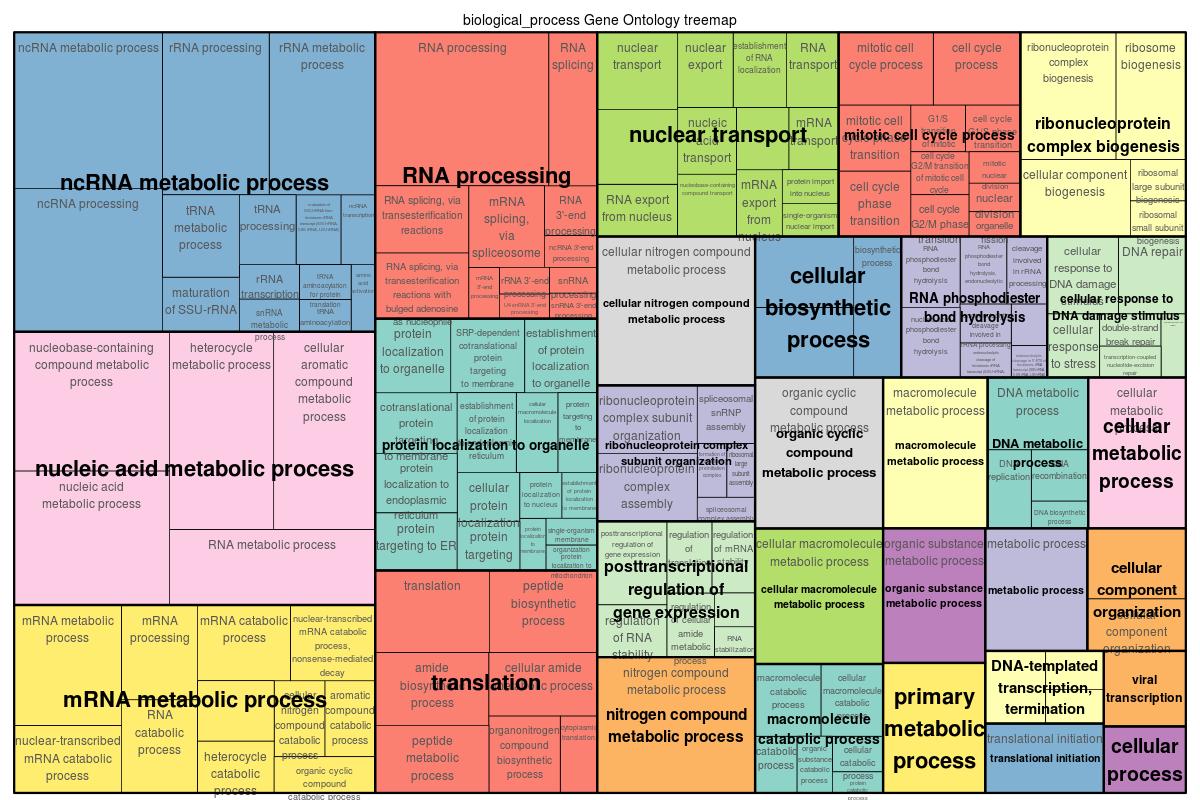


**Supplement Figure SM3**

Enriched TRANSPATH Pathways (2017.2) of upregulated genes in Cancer vs. Control.


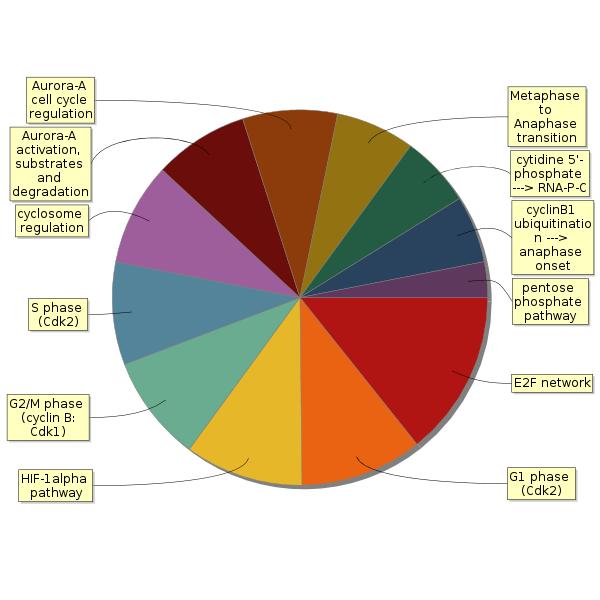


**Supplement Figure SM4**

Enriched HumanPSD(TM) disease of upregulated genes in Cancer vs. Control.


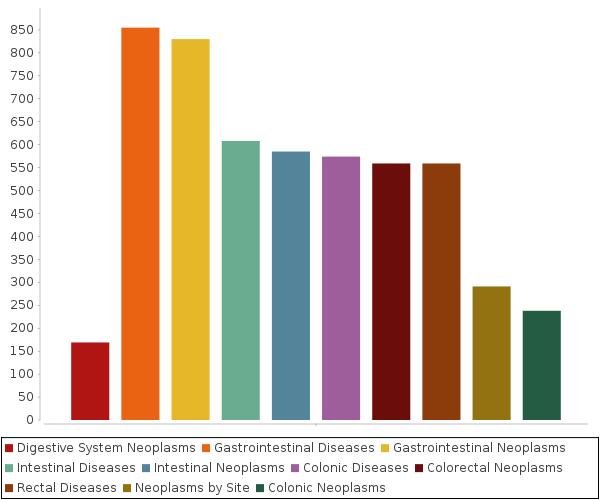


**Supplement Figure SM5**

Scheme of the location of methylation sites, correlated with the expression of the genes in which they are located. The scheme is generated by the web tool My Genome Enhancer (my-genome-enhancer.com) (see description in the Method section). Genes with increased expression in stage-I tumors of colorectal cancer are selected here. The blue rectangles show the exons of the genes, the arcs show the introns of the genes, straight lines show the 5 'and 3' non-coding regions of the genes. The introns and non-coding regions are shown in logarithmic scale. Red vertical lines show the positions of methylated CpG dinucleotides.


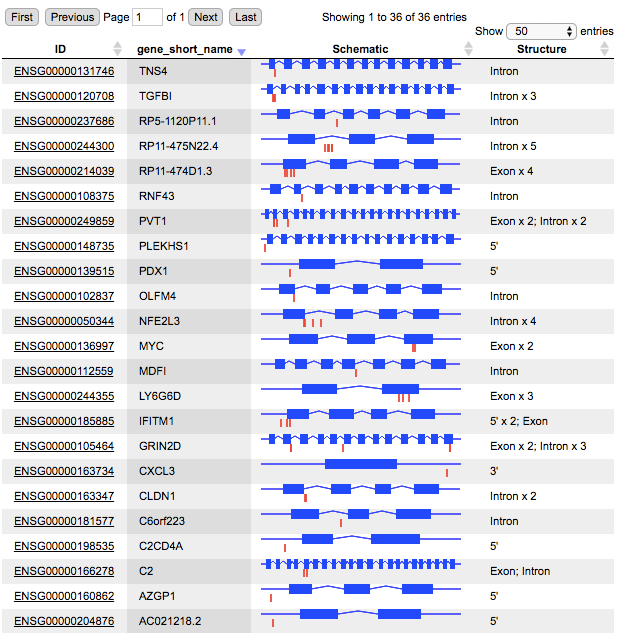


**Supplement Figure SM6**

A screenshot from the geneXplain platform with detailed information about composite modules that were found in the NEG CpG methylated regions (449 regions) and also the statistical parameters of the constructed model. In the histogram at the left there is a distribution of the score values of the composite module in the Yes set of genomic regions (red) and distribution of the score values in the background set of genomic regions (No set). At the right panel there is a list of PWM with their optimized cut-offs that contribute to the composite model. The statistical parameters of the model, like AUC (AUC=0.77) shows good discrimination of Yes and No test of regions.


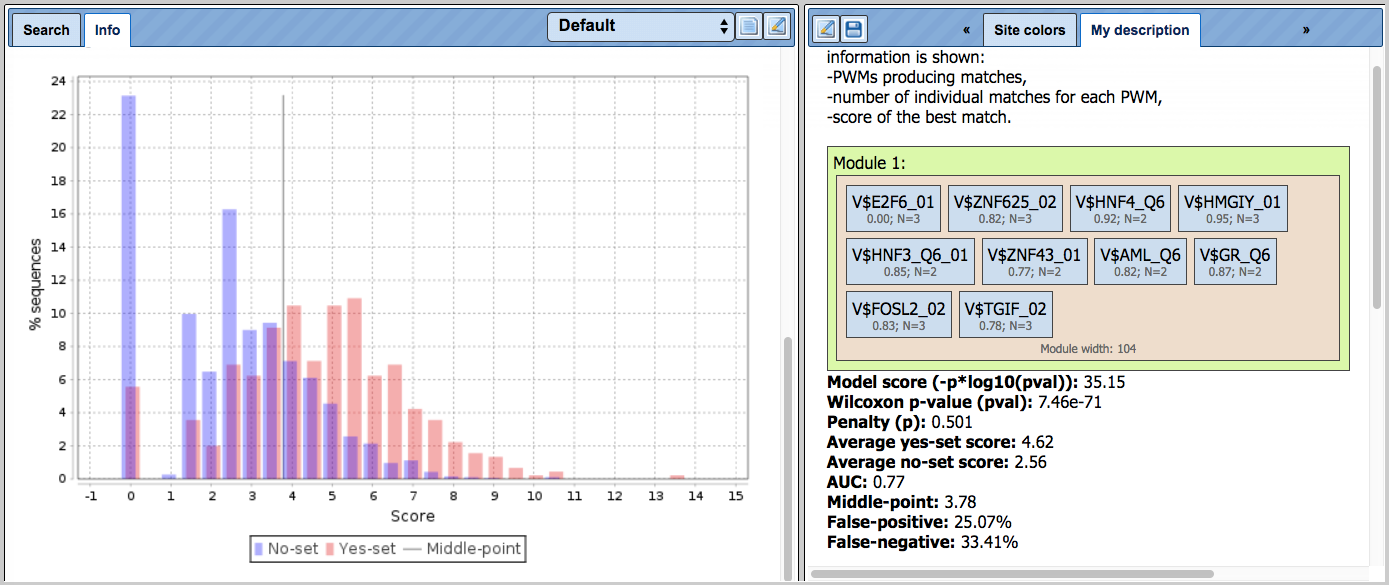


(the picture in a separate file: Additional file 13 - Fig_S5_CMA_NEG.tiff)

**Supplement Figure SM7**

A screenshot from the geneXplain platform with detailed information about composite modules that were found in the POS CpG methylated regions (339 regions) and also the statistical parameters of the constructed model. In the histogram at the left there is a distribution of the score values of the composite module in the Yes set of genomic regions (red) and distribution of the score values in the background set of genomic regions (No set). At the right panel there is a list of PWM with their optimized cut-offs that contribute to the composite model. The statistical parameters of the model, like AUC (AUC=0.87) shows pretty good discrimination of Yes and No test of regions.
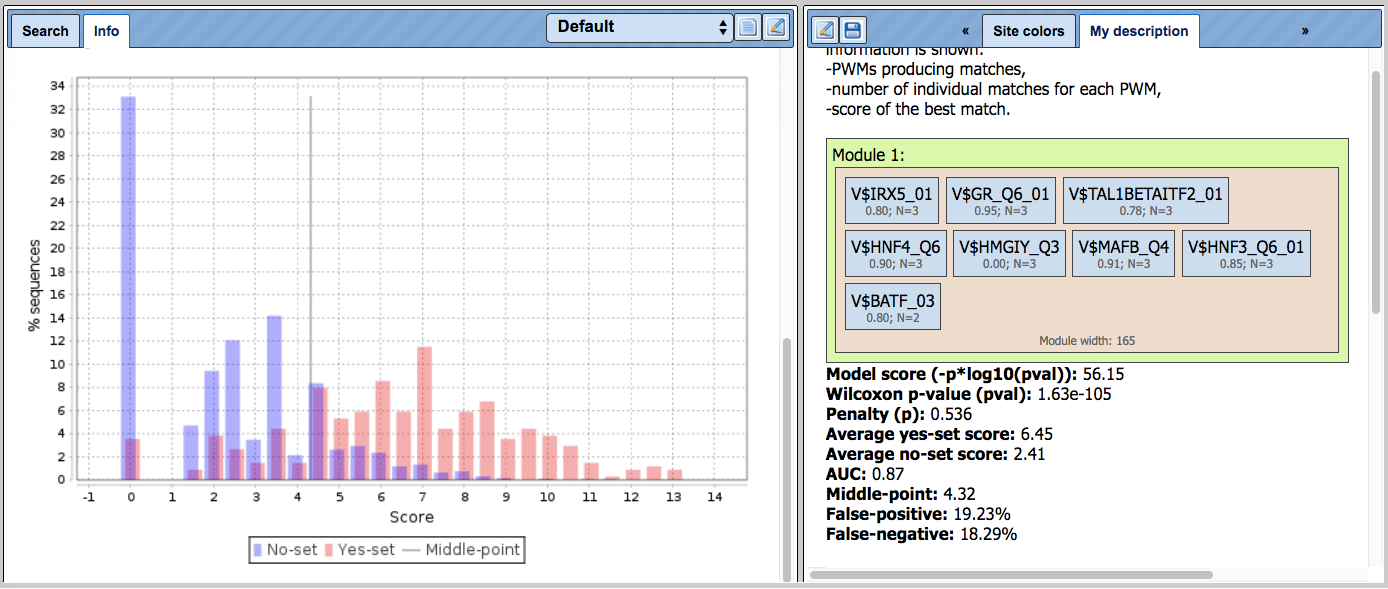


(the picture in a separate file: Additional file 14 - Fig_S6_CMA_POS.tiff)

**Supplement Figure SM8**

The web interface of the diagnostic tool. User inputs DNA methylation values of 6 biomarkers for one or more samples and the tool makes a classification of the samples as CRC or non-CRC. It is accessible through the following URL: ([http://micro.biouml.org/bioumlweb/#anonymous=true&perspective=DNA%20methylation%20signature%20for%20the%20diagnosis%20of%20colon%20cancer](http://micro.biouml.org/bioumlweb/#anonymous=true&perspective=DNA%20methylation%20signature%20for%20the%20diagnosis%20of%20colon%20cancer))


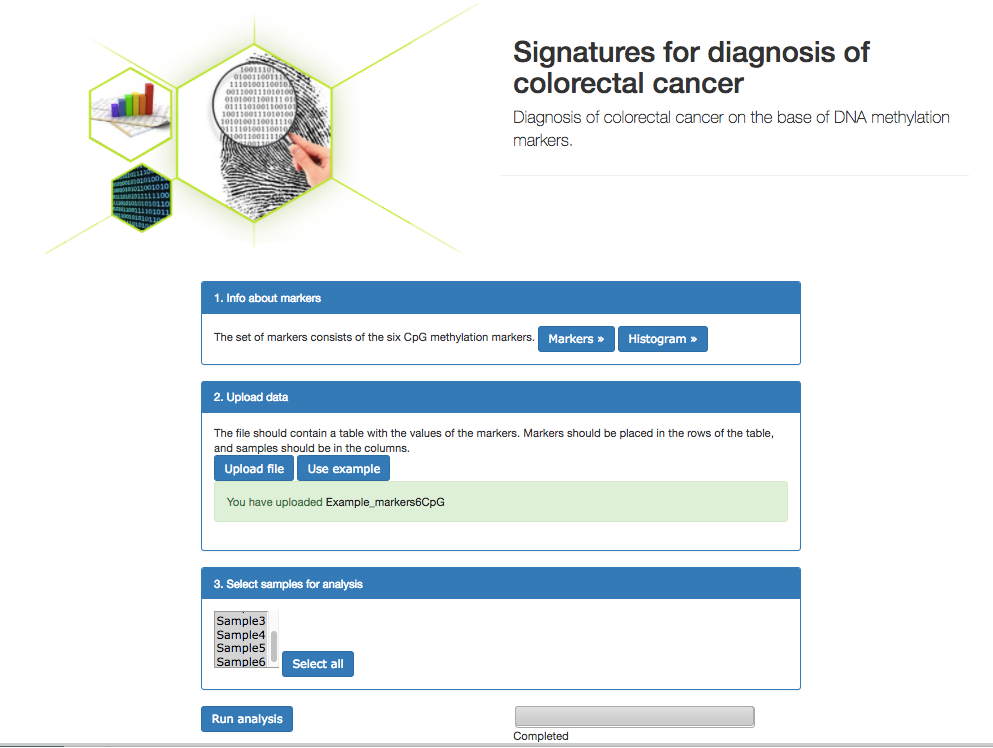

Supplement: Supplementary file 3 — An MS Office file (.docx format) that contains Table S12 and Figures S2–S8. (DOCX 1437 kb) [file 12859_2019_2687_MOESM3_ESM.docx]
